# Challenges in the use of sortase and other peptide ligases for site-specific protein modification

**DOI:** 10.1039/d0cs01148g

**Published:** 2022-05-05

**Authors:** Holly E. Morgan, W. Bruce Turnbull, Michael E. Webb

**Affiliations:** School of Chemistry and Astbury Centre for Structural Molecular Biology, University of Leeds, Woodhouse Lane, Leeds LS2 9JT UK w.b.turnbull@leeds.ac.uk m.e.webb@leeds.ac.uk

## Abstract

Site-specific protein modification is a widely-used biochemical tool. However, there are many challenges associated with the development of protein modification techniques, in particular, achieving site-specificity, reaction efficiency and versatility. The engineering of peptide ligases and their substrates has been used to address these challenges. This review will focus on sortase, peptidyl asparaginyl ligases (PALs) and variants of subtilisin; detailing how their inherent specificity has been utilised for site-specific protein modification. The review will explore how the engineering of these enzymes and substrates has led to increased reaction efficiency mainly due to enhanced catalytic activity and reduction of reversibility. It will also describe how engineering peptide ligases to broaden their substrate scope is opening up new opportunities to expand the biochemical toolkit, particularly through the development of techniques to conjugate multiple substrates site-specifically onto a protein using orthogonal peptide ligases.

## Abstract 2

We highlight chemical and biochemical strategies taken to optimise peptide and protein modification using peptide ligases.

## Graphical abstract

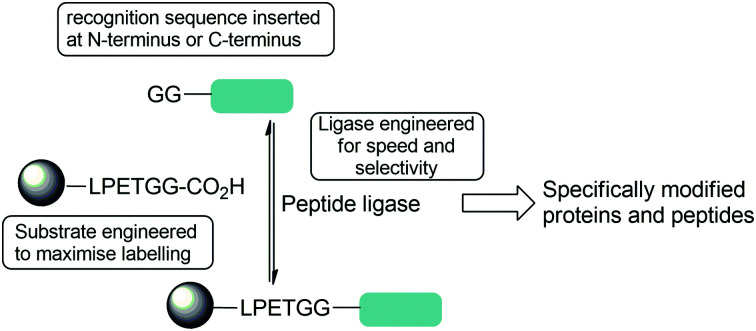

## 1 Introduction

Site-specific protein modification is widely used for a range of applications including the production of biopharmaceutical products and the investigation of protein function in living systems. Since traditional chemical protein modification methods use reactions of naturally-occurring amino acid functional groups found in the protein, acheiving site-specificity is difficult.^1,2^ Incorporation of non-natural amino acids with bioorthogonal groups into a protein can address this problem, but this can involve extensive modification of the protein expression conditions to generate the modified substrate protein.^3,4^ Exploitation of enzymes which modify proteins is therefore an attractive option; their main advantages are their inherent specificity and usually mild reaction conditions. There are a wide range of such enzymes and reactive protein domains including, for example formylglycine generating enzyme,^5^ SpyTag^6^ and SNAPTag^7^ in which defined sequences and domains can be post-translationally modified however peptide ligases are unique in their ability to catalyse the formation of peptide bonds, allowing the natural protein backbone to be preserved.^8^ The recognition sequences are typically small and this makes them particularly attractive for protein engineering purposes. The capabilities of peptide ligases to form defined complexes in high yields means that are now increasingly used to generate complex engineered proteins *in vitro*, including antibody drug-conjugates, site-specifically modified histones and proteins with defined ubiquitinylation states; and as tools *in vivo* to selectively modify particular proteins in the cell, on the cell surface and in plasma.

**Scheme 1:**
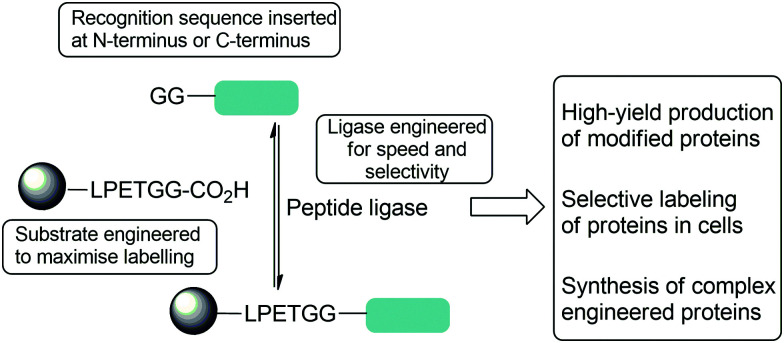
Summary of strategies used to optimise reactions of peptide ligase to enable complex protein modification reactions including both substrate and protein engineering described in this review.

**Scheme 2:**
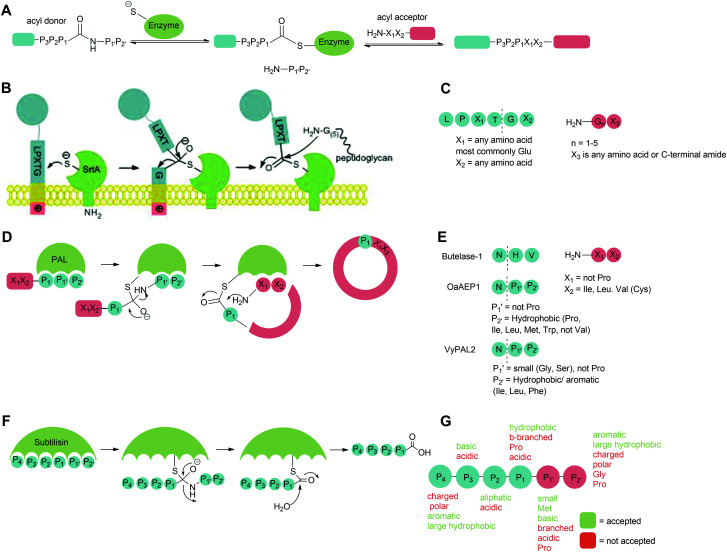
Catalytic mechanism of (A) peptide ligases, (B) Sortase A on the surface of Gram-positive bacteria, (D) peptide asparaginyl ligases (PALs) to produce cyclic peptides in plants, (F) subtilisin in *Bacillus amyloliquefaciens*. Substrate specificity of (C) Sa Sortase A, (E) PALs and (G) subtiligase.

This review will explore the key examples of peptide ligases used for protein modification, focusing mainly on sortase, the leading enzyme in the field. The peptidyl asparaginyl ligases Butelase-1, OaAEP1, VyPAL2 and peptide-ligating variants of subtilisin will also be discussed (Scheme 2). The challenges associated with this approach to protein modification will be highlighted, and how engineering of peptide ligases and their substrates has been used to address these challenges. The three principal challenges in developing new methods are ensuring specificity, efficiency and versatility: that modification is site-specific and generates well-defined conjugates; that it is time and reagent efficient; and that it is versatile (Scheme 1). We will first discuss each class of enzyme from the perspective of engineering enhanced catalytic activity. The review will then focus on examples of substrate engineering that aim to reduce the reversibility of the ligation reaction, and thus drive conversion of substrates to products. Studies that have broadened substrate specificity will then be presented, before the final section of the review illustrates how these advances have created new opportunities in the field of protein modification; in particular, the use of orthogonal peptide ligases to conjugate multiple substrates site-specifically onto a protein.

## 2 Peptide ligases and enzyme engineering to enhance catalytic activity

Peptide ligases catalyse the formation of an amide bond, usually at the N- or C-terminus of a peptide or protein substrate. The reaction mechanism typically proceeds *via* cleavage of a recognition sequence at the C-terminus of a peptide/protein by a cysteine residue to form a peptide/protein acyl–enzyme intermediate (Scheme 2A). Nucleophilic attack on the acyl intermediate by an N-terminal amine (aminolysis) in the second substrate releases the enzyme and results in the formation of a peptide bond. Whilst limited examples of such peptide ligases exist in nature, proteases which catalyse the hydrolysis of peptide bonds are much more abundant. While the mechanism of serine and cysteine proteases also involves an acyl–enzyme intermediate formed by the catalytic nucleophile, aminolysis is inefficient and the intermediate is instead hydrolysed. Efforts have therefore been made to engineer proteases into ligases by altering the catalytic mechanism in order to increase the ratio of aminolysis to hydrolysis.^9^ The development of enzymatic protein modification techniques has been driven by this kind of enzyme engineering, with the objective of improving the catalytic efficiency of established ligases as well as altering the behaviour of such proteases. As described below, this has led both to enhanced reaction rates and a concomitant reduction in the amount of catalyst required, both of which are desirable qualities in a protein modification technique.

### 2.1 Sortase

Sortases are a class of transpeptidase enzymes that covalently attach an array of proteins to the surface of Gram-positive bacteria.^10^ Sortases can be divided into six distinct families (A–F) on the basis of structure and substrate dependence.^11–13^ The sortase A family is best characterised, and members of this family are present in almost all Gram-positive bacteria.^14^ This class of sortase enzymes performs a housekeeping role in the bacterial cell, anchoring a large number of functionally-distinct proteins to the cell wall. The sortase that has been studied most extensively is *Staphylococcus aureus* Sortase A (SaSrtA), which acts upon proteins with a C-terminal LPXTG recognition motif (Schemes 2B and C; where X denotes any amino acid.).^12,15^ Upon binding of the recognition motif in the catalytic site, the sulfhydryl group of Cys184, as part of a catalytic triad with His120 and Arg197, attacks the backbone carbonyl of the threonine residue in the LPXTG, cleaving the threonine-glycine bond and forming a thioester intermediate (Scheme 2B). This intermediate is then attacked by the N-terminal amine of a pentaglycine motif in peptidoglycan, releasing the enzyme and covalently linking the protein to the cell wall.^12,14,16–19^ The catalytic activity of SaSrtA is facilitated by the binding of calcium ions into a binding pocket located near the active site.^20^ The resulting structural change in the active site supports favourable interactions with the LPXTG motif.^14^ This dependence on calcium is specific to SaSrtA. The residues involved in binding Ca^2+^ are not conserved in other Gram-positive bacterial sortase A enzymes such as those from *Bacillus anthracis* SrtA (BaSrtA) and *Streptococcus pyogenes* (SpSrtA).^21,22^

The activity of sortase has been extensively exploited to perform protein/peptide protein modification. This strategy requires purified SaSrtA, a donor substrate containing the C-terminal LPX_1_TGX_2_ recognition motif and an acceptor molecule with a sterically-unhindered (N-terminal glycine residue). While the recognition sequences for sortases are typically given in the literature in the form LPXTG and are used in this review for clarity, in general, the required recognition motif is LPX_1_TGX_2_ (Scheme 2C) where X_2_ is either a C-terminal amide or another amino residue; protein or peptides where the glycine nucleophile has a free carboxylic acid group are not substrates for sortases. In the authors’ experience, this additional requirement is frequently overlooked by those using sortases for the first time. For C-terminal protein modification, an LPXTG recognition motif is required at the C-terminus of the protein and the substrate to be ligated must contain an N-terminal glycine residue. The accessibility and flexibility of both the N- and C-terminal region impacts the efficiency of the reaction.^23–25^ One downside to C-terminal labelling is that the LPXTG sequence must be engineered into the protein. Applications of this method are also limited for modification of cell surface proteins which most commonly have intracellular C-terminal regions and extracellular N-terminal regions, and thus cannot be labelled *via* this method.^26^ Alternatively, N-terminal protein labelling involves ligation of a labelling substrate with a C-terminal LPXTG motif to a protein with an N-terminal glycine.^25^ It requires minimal engineering of the protein, only requiring a single N-terminal glycine in a sterically unhindered position. Many commercial expression plasmids have N-terminal protease recognition sequences that, when cleaved, result in a protein that already possesses an N-terminal glycine.^27,28^ There is also potential for internal labelling of a protein by introducing a flexible loop into the protein.^23^ Guimaraes *et al.* demonstrated a method where a loop, containing the LPXTG recognition motif followed by a specific protease cleavage site, was introduced between two cysteine residues which formed a disulfide bond in the protein. The flexibility of the loop was increased by nicking the loop with a protease, allowing the sortase-mediated reaction to occur as it would for a C-terminal labelling reaction. If the loop is flexible and accessible, proteolysis may not be required.

Over the years, sortase-mediated ligation has proven itself to be a key protein conjugation technique. It has been used for a variety of applications including protein–protein fusion,^29,30^ protein cyclisation,^31–33^ immobilisation of proteins onto artificial surfaces^34,35^ and introducing novel functionality, such as fluorescent tags,^36^ peptides,^37^ lipids^38^ and toxins^39^ into proteins site-specifically. However, it does possess some limitations and a significant amount of work has been carried out to increase the catalytic efficiency, eliminate the dependence on calcium ions, increase the rate of transpeptidation and reduce the rate of hydrolysis and reaction reversal. Many of these challenges have been addressed through enzyme engineering.

#### 2.1.1 Expression and purification of sortase

Ton-That *et al.*^17,40^ originally produced recombinant SaSrtA enzymes (SrtAΔ59 and SrtAΔ25) by removing the N-terminal membrane-anchoring segment of the protein and replacing it with a His_6_ tag. This enabled the expression of a soluble enzyme and purification by nickel affinity chromatography and was instrumental for the widespread use of sortase for protein modification. In addition to aiding purification of sortase after expression, purification tags are also useful for removing sortase from a labelling reaction. This is advantageous as it can prevent hydrolysis, reversal of the labelling reaction and facilitates immobilisation in flow channels. Other purification tags, such as chitin binding domain and maltose binding protein have also been reported and applied.^28,41^ Sortase A has been cloned and is available from plasmid repositories with *e.g.* C-terminal His-tags, enabling its ready adoption.^19,42,43^ In our own experience, sortases of all types are readily overexpressed in high yield and show high stability compared to most other recombinant proteins; their supply is therefore not a limiting factor.

#### 2.1.2 Enhancement of catalytic activity

**Fig. 1:**
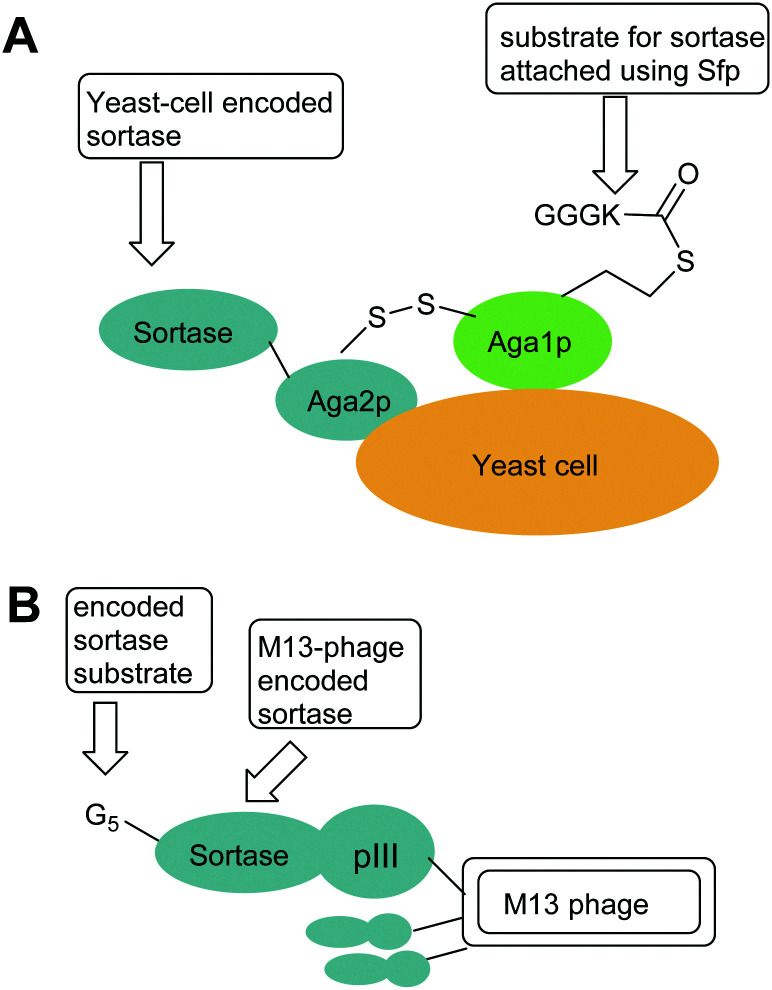
Exemplar yeast and phage constructs used for directed evolution of sortases. In both cases, sortases are encoded by phage or yeast cells and the activity of the encoded sortase is probed by addition of a biotinylated sortase substrate (*e.g.* Biotinyl-LPETGG) which enables isolation of phage of yeast encoding active sortases. (A) Aga1p–Aga2p strategy used by Chen *et al. i* to increase sortase activity.^44^ (B) M13 Phage strategy used by Piotukh *et al* to identify sortases with altered specificity.^45^

**Fig. 2:**
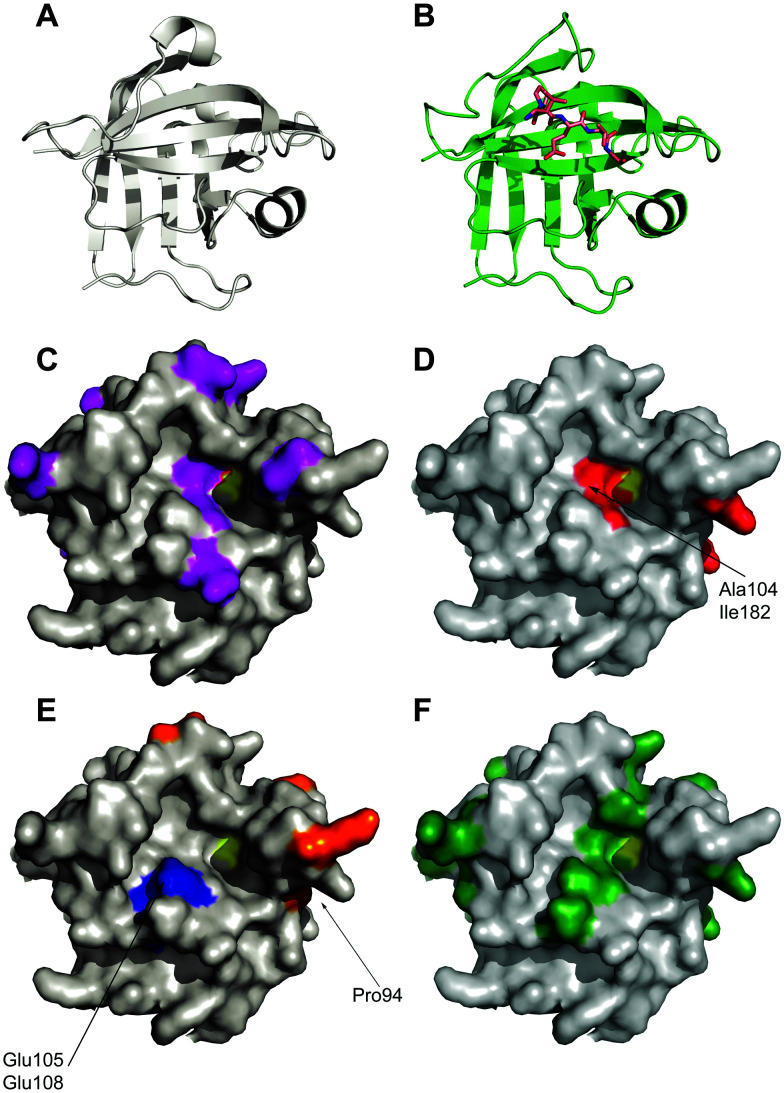
Location of mutations in sortase variants mapped onto the crystal structure of Sortase A. (A) Apo-crystal structure of WT sortase A determined by Zong *et al.* (1t2p)^18^ (B) structure of a LPETG peptide bound to Sortase A. (1t2w) (C) location of mutations observed in eSrtA(2A-9) shown in purple.^64^ Active site cysteine yellow. (D) Location of mutations observed in eSrtA(4S-9) shown in red.^64^ (E) Location of mutations observed in SrtA(5M)^44^ (orange) and SrtA(7M)^52^ (orange and blue). (F) Location of mutations found in SrtAβ (dark green).^65^

Wild-type SaSrtA catalyses ligation reactions relatively poorly, (*k*_cat_/*K*_M LPETG_ = 200 M^−1^ s^−1^). In practical terms this means that large amounts of catalyst and prolonged reaction times are required for complete reaction. Therefore, in 2011, Chen *et al.*^44^ developed a directed evolution technique integrating yeast display, enzyme-catalysed small molecule–protein conjugation and fluorescence-activated cell sorting (FACS) to evolve SaSrtA for improved catalytic activity. They initially focused on decreasing the high *K*_M_ for the LPXTG substrate (7.6 mM). WT SaSrtA was subjected to mutagenic PCR before subcloning into a yeast display vector. The resulting modified proteins (library size ∼8 × 10^7^) were displayed on the yeast cell surface as fusion proteins with the cell surface mating factor Aga2p. This protein forms a disulfide-linked dimer with the protein Aga1p. In order to enable screening this protein was first modified using Sfp-mediated linkage of either CoA-LPETGG or GGGK(CoA) substrates to a 12-residue S6 recognition peptide added to its N-terminus (Fig. 1A). Yeast cells containing active sortase could therefore catalyse the coupling of these Aga1p-linked substrates to complementary biotinylated substrates in solution in a pseudo-intramolecular reaction, linking the biotin handle to the yeast surface enabling selection of active mutants. To drive selection of mutants with higher affinity, the concentration of Biotinyl-LPETGG in solution was reduced through several rounds of selection including a second round of mutagenesis. Sequencing of the evolved sortase genes in the final round revealed the predominance of P94S or P94R, D160N, D165A, and K196T mutations. Combination of all four mutations yielded an enzyme with a 140-fold increase in *k*_cat_/*K*_M_(LPETG) compared to WTSrtA. Further mutagenesis and two rounds of directed evolution yielded a pentamutant (P94R/D160N/D165A/K190E/K196T) termed evolved sortase A (eSrtA or Srt5M see Table 1 and Fig. 2). eSrtA has a 120-fold higher *k*_cat_/*K*_M_(LPETG) compared to WTSrtA, as well as a 20-fold lower *K*_M_ for the polyglycine second substrate and was shown to be substantially more effective than the WTSrtA at labelling LPETG-tagged proteins on the surface of live mammalian cells.

**Table 1:** A subset of reported sortase varients derived from wild-type and evolved sources including sortases with enhanced catalytic activity (*e.g.* eSrtA), Ca-independence *e.g.* SrtA(7M), altered substrate specificity *e.g.* eSrtA(4S-9) and SrtAβ, and enhanced thermal stability

Sortase	Recognition sequence	Ref.	Notes
**Wild-type sortases**			
			
SaSrtA (*Staphylococcus aureus*)	LPXTG	Ton-That *et al.*^40^	Anchors protein to the cell wall *in vivo*, Poor kinetics *in vitro* Calcium dependent
			
SrtB^58^ (Bacilli, Listeria and *S. aureus*)	NPQTN	Mazmanian *et al.*^58^	Found in the iron-responsive determinant locus (involved in iron acquisition, important in bacterial pathogenesis). Anchors IsdC to the cell surface
SrtC^59^ (Actinomyces, Corynebacteria, Enterococci and Streptococci)	QVPTG	McCafferty & Melvin^59^	Polymerisation of pilin proteins
SrtD^60^ (sporulating Gram-positive bacteria)	LPNTA	Marrafini & Schneewind^60^	Responsible for targeting BasH and BasI in sporulating bacilli
SpSrtA^21^	LPXTG/LPXTA	Race *et al.*^21^	Calcium independent
BaSrtA	LPXTG	Weiner *et al.*^22^	Calcium independent
SavSrtE	LAXTG/LPXTG	Das *et al.*^13^	Calcium independent
CdSrtA	LPLTG	McConnell *et al.*^61^	Generates an isopeptide bonds to Lys in WxxxVxVYPK̲ motif in pilin

**Sortases with enhanced catalytic activity**			
			
eSrtA (SrtA(5M))P94R/D160N/D165A/K190E/K196T	LPXTG, LPEXG (X = A, C, S) LAETG	Chen *et al.*^44^	Evolved from SaSrtA
			Improved kinetics
SrtA(5M/Y187L/E189R) SrtA(5M/D124G	LPXTG	Chen *et al.*^46^	Evolved from SaSrtA and SrtA(5M) Improved reaction for N- and C-terminal labelling respectively
			
E105K/E108A/Q mutant	LPXTG	Hirakawa *et al.*^50^	Evolved from SaSrtA Calcium-independent
			
SrtA(7M) P94R/E105K/E108Q/D160N/D165A/K190E/K196T	LPXTG	Wuethrich *et al.*^52^	Evolved from SaSrtA Improved kinetics, calcium independent

**Sortases with altered specificity**			
			
SrtLS SaSrtA β6/β7 loop exchanged for SaSrtB β6/β7 loop	NPQTN	Bentley *et al.*^62^	Evolved from SaSrtA Only catalyses acylation, not transpeptidation
			
F40-sortase T164Q/V168M/L169H/D170L/E171A/Q172E	XPKTG (X = A, D, S), APATG	Piotukh *et al.*^45^	Evolved from SaSrtA
F1-21 sortases V161Y/K162W/P163A/T164N/D165E/V166R/G167I/V168F/L169H/D170V/E171L	APXTG/FPXTG	Schmohl *et al.*^63^	Evolved from SaSrtA
eSrtA(2A-9) S102C/A104H/E105D/K138P/K152I/N160K/K162H/T164N/K173E/I182V/T196S	LAETG	Dorr *et al.*^64^	Evolved from SrtA(5M)
eSrtA(4S-9) N98D/S102C/A104V/A118T/F122A/K134R/F144L/I182V/E189F	LPEXG (X = A, C, S)	Dorr *et al.*^64^	Evolved from SrtA(5M)
SrtAβ I76L/S102C/E105D/N107E/S118I/I123L/D124L/N127H/G134R/K138L/G139D/M141I/K145T/K152R/M155I/R159C/K162R/Q172H/K73E/K177R/V182A/V189Y/T196S/R197S/K206R	LMVGG	Podracky *et al.*^65^	Evolved from 4S-6 (LPESG-specific)
			
SpSrtA M3 E189H/V206I/E215A	LPXTG,	Zou *et al.*^66^	Recognises N-terminal GG, AA, SS and CC substrates Evolved from SpSrtA

**Sortases with increased stability**			
			
SaSrtA rM4 P94S/D160N/D165A/K196T	LPXTG	Zou *et al.*^67^	Evolved from SaSrtA higher activity than WT at ambient temperature but lower thermal stability, resistant to DMSO
			
SaSrtA CyM6 P94S/D160N/D165A/K196T R159N and K162P Head to tail cyclisation	LPXTG	Zou *et al.*^68^	Evolved from SaSrtA (through rM4) Improved thermostability and resistance to chemical denaturation

Further improvements in efficiency over eSrtA have been obtained using a FRET screening approach.^46^ In this case, as well as an error-prone PCR-based approach on the whole enzyme, site-saturated mutagenesis at a set of rationally selected sites on WTSrtA or eSrtA was employed. The libraries were screened using a sortase ligation-dependent FRET pair of eGFP-LPETG and GGG-cpVenus. In particular, the 5M/Y187L/E189R variant was found to be highly effective for C-terminal antibody modifications and the 5M/D124G variant was superior for N-terminal antibody modification (Table 1).

Another strategy for sortase evolution, SortEvolve, was reported by Zou *et al.*^47^ This approach, which uses a high-throughput screening platform in microtitre plate format, was validated by the same range of mutations. In this case, sortase mutants mediate fusion of the laccase CueO with a C-terminal LPETGGGRR tag to GGG-eGFP-LCI. The degree of ligation was then assayed by LCI-mediated immobilisation of the fusion product to polypropylene plates and assay of laccase concentration using 2,2′-azino-bis(3-ethylbenzothiazoline-6-sulfonic acid (ABTS)). To validate this system, three site-saturated mutagenesis (SSM) SaSrtA libraries were generated at three positions (P94, D160, and D165). Each SSM-library was screened independently in one 96-well microtitre plate. The previously reported P94S, D160N, and D165A mutants were identified. Further recombinant Sa-SrtA variant P94T/D160L/D165Q was characterised with 22-fold improvement in catalytic efficiency compared with the wild-type protein.

More recently, Li *et al.*^48^ have investigated the behaviour of intermediate sortase variants in which only a subset of these mutations are included and highlighted that some of these appear to be optimal for a different range of applications. It was determined that each variant has advantages appropriate for specific applications when considering rate of reaction, extent of hydrolysis, purification restraints, temperature, and additives *e.g.*, detergent requirements.

#### 2.1.3 Calcium dependence

The second limitation of SaSrtA is the requirement for calcium to stabilise the active site.^20^ The Ca^2+^ dependency of SaSrtA limits its application for protein modification as it is difficult to use under low Ca^2+^ concentrations, such as in living cells, and in the presence of Ca^2+^ binding substances, such as buffers containing phosphate, carbonate or chelators like EDTA.^31^ One solution to this problem is to use naturally calcium-independent sortases such as demonstrated by Strijbis *et al.*,^49^ who used calcium-independent SpSrtA to modify proteins inside *S. cerevisiae* and in mammalian HEK-293T cells. However, the specific activity of SpSrtA is much lower than SaSrtA. Another solution was found through the development of a calcium independent SaSrtA variant. In WT SaSrtA, calcium ions bind to the calcium binding pocket by forming interactions with residues Glu105, Glu108 and Glu171 in the β3–β4 loop.^20^ This allows the unstructured and flexible β6–β7 loop to adopt a closed conformation in which Val166, Val168 and Leu169 can bind the LPXTG motif in the active site.^14^

An alternative approach was reported by Hirakawa *et al.*^50^ who used a structure-guided alignment of SaSrtA with the calcium-independent enzymes SpSrtA and BaSrtA in order to develop SaSrtA variants with Ca^2+^-independent catalytic activity. This indicated that Glu105 and Glu108, are not conserved in SpSrtA or BaSrtA. In SpSrtA, Glu105 corresponds to Lys126 which forms a salt bridge with Asp196 (Glu171 in SaSrtA) which may stabilise the closed conformation of the β6/β7 loop instead of calcium ions. In SaSrtA, Glu105, Glu108 and Glu171 coordinate to Ca^2+^.^51^ Hirakawa therefore hypothesised that substitution of Glu108 with an uncharged amino acid, together with substitution of Glu105 with Lys, would moderate the negative charge concentrated in the calcium binding site and overcome the calcium dependency of SaSrtA. Consequently, both double mutants E105K/E108A and E105K/E108Q were shown to enhance protein ligation in the absence of calcium, without drastically affecting substrate specificity(see Fig. 2). Overall, however the calcium-independent activity of these proteins was ∼65% lower than the calcium-dependent activity of the WT SaSrtA.

The Ploegh group combined the eSrtA pentamutant with the second of these calcium-independent variants to create the heptamutant SrtA(7M).^52^ This has a 40-fold higher *k*_cat_/*K*_M LPETG_ compared with the double mutant (E105K/E108A). Thus, as a result of these mutations, a catalytically efficient, calcium-independent sortase enzyme was evolved(see Fig. 2 and Table 1). Despite obvious advantages with the use of the pentamutant and heptamutant, these enzymes are not optimal for all applications as they are prone to higher levels of hydrolysis if not carefully monitored.^51^ Different variants are suitable for different applications, as made evident by Li *et al.*^48^ who have subsequently evaluated the use of SaSrtA variants 3M, 4M and 5M for a range of ligation reactions.

#### 2.1.4 Increasing catalytic efficiency

**Scheme 3:**
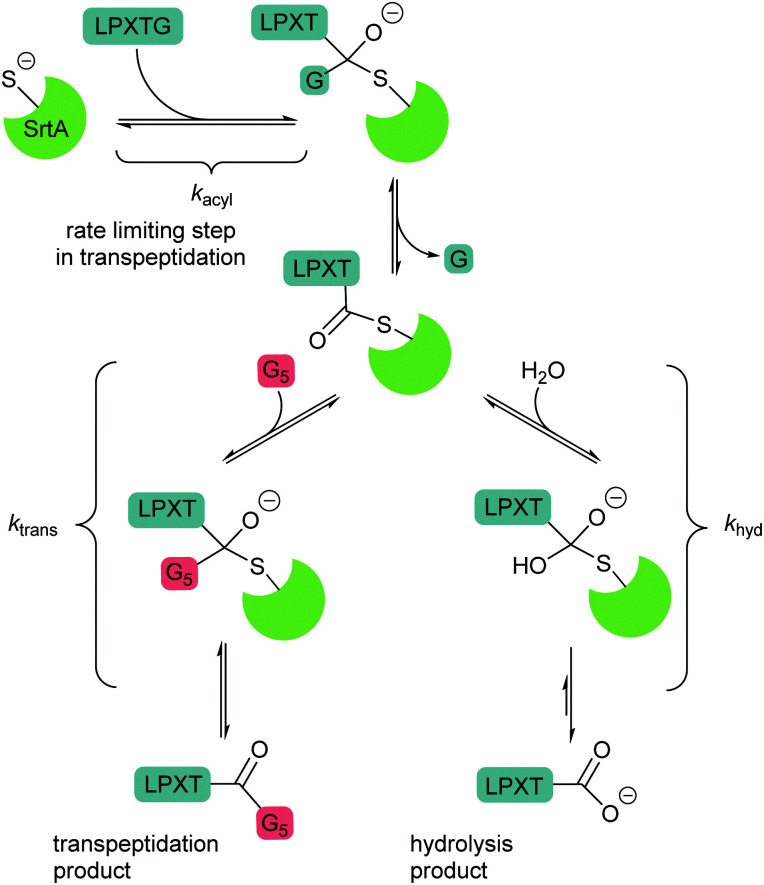
Mechanisms of competing transpeptidation and hydrolysis reaction. In the absence of an acyl acceptor substrate, the by-product peptide reversibly forms the starting material competitively inhibiting the hydrolysis reaction.

Huang *et al.*^53^ and Frankel *et al.*^54^ (Scheme 3) have both studied the kinetics of each step of the sortase-catalysed reaction – both found that at the optimal pH, the transpeptidation reaction is limited by initial acylation of the enzyme (binding of the recognition motif to the sortase catalytic region), whereas hydrolysis of the acyl–enzyme intermediate is the rate-limiting step in the hydrolysis reaction. Kinetic studies have revealed that the *k*_hyd_ for hydrolysis is much slower than aminolysis (the transpeptidation reaction).^54^ Partitioning of the thioacyl intermediate towards hydrolysis rather than aminolysis is more likely to take place when the concentration of the oligoglycine substrate is low or at pH below the p*K*_a_ of the N-terminal amine (∼pH 8). An optimum in the overall rate was observed at around this pH by Wu *et al.*,^55^ consistent with this observation. However, the product of transpeptidation can reform the thioacyl intermediate and, over time, the irreversible hydrolysis product can therefore be formed. The rate-limiting nature of thioacyl intermediate formation means that long reaction times are generally required. While enzymes with enhanced catalytic activity, *e.g.*, SrtA(7M), have decreased the reaction times and the concentrations of sortase and substrate required, these are accompanied by an increase in the rate of hydrolysis which particularly needs to be controlled for C-terminal labelling. Optimisation of such reactions is generally needed to ensure that hydrolysis does not occur upon over-long incubation. Several different enzyme-based strategies have been adopted to increase the efficiency of these labelling reactions including proximity-based labelling and flow-based approaches. Two different groups have reported the covalent fusion of sortase to one or other substrate to enhance reaction rate. Amer *et al.*,^56^ created a fusion between sortase and SUMO (small ubiquitin-like modifier protein) as a solubility tag with an N-terminal pentaglycine motif. Reaction of this fusion tag with an isotopically labelled substrate containing an LPXTG motif led to enhanced reaction relative to the intermolecular reaction of the separate components. This approach has the disadvantage that the sortase remains covalently linked to the protein after the reaction. Alternatively, a traceless proximity-based approach using SpyTag/SpyCatcher has been used to link the sortase to the LPXTG substrate motif.^57^ In this case, the target protein, has an additional C-terminal SpyTag sequence (a 13 aa peptide) after the LPETG sortase recognition motif. A resin-immobilised SpyCatcher-SaSrtA fusion protein was then used to capture the protein-SpyTag fusion *via* formation of an irreversible isopeptide bond to the SpyCatcher domain. This brings the protein into close proximity with SaSrtA and the ligation reaction can then be initiated *via* the addition of calcium ions and peptide with an N-terminal GGG. This ligation reaction leads to cleavage of the labelled protein from the resin (however hydrolysis is still a possibility in this system). Witte *et al.*^69^ used a contrasting immobilised approach by immobilising sortase on resin, flowing over the LPETG-containing reactant to generate the immobilised reactant before incubation of the immobilised thioacyl intermediate with the nucleophilic acyl donor. By removing the original glycinyl leaving group from the system prior to addition of the second substrate, an equilibrium mixture of the LPETG-containing reactant and product is avoided.

#### 2.1.5 Increased stability

SaSrtA is inactivated at elevated temperatures and in the presence of denaturing agents, which limits its application in immobilised enzyme applications where the catalyst will be reused repeatedly and in peptide ligations. Zou *et al.*^68^ used loop engineering and head-to-tail backbone cyclization to increase the stability of the enzyme SaSrtA. Initial work was based on a DMSO-resistant mutant SaSrtA rM4 (Table 1), which has a 45-fold improved LPETG recognition and a 3-fold gain in *k*_cat_ (140-fold increase in catalytic efficiency) compared to WT at ambient temperature but low thermal stability.^44,67,70^ Protein fragment ligation of P_450_ BM3 monooxygenase was used to assay sortase activity in a high-throughput screening approach. Two key variants in the flexible β6/β7 loop, rM4-K162P and rM4-R159N, were identified which displayed 3.5-fold and 3.0-fold increased activity, respectively. These increases may be attributed to enhanced hydrophobic interactions towards the LPETG substrate. Additionally, a 3.1-fold increased thermal stability was also seen in rM4-K162P, mostly likely due the proline ring limiting the conformational mobility of SaSrtA. Consequently, the R159N and K162P mutants were combined to produce SaSrtA M6, which showed a further 8-fold increase in activity and a 5-fold increase in thermal stability compared to rM4. Finally, SpSrtA was used to catalyse head-to-tail backbone cyclisation to produce a cyclic hexamutant, CyM6. This construct retained 99% of activity and had a 7.5 °C increase in thermal stability relative to rM4, significantly enhancing storage stability compared to WT. This form of the enzyme showed significant increase in activity (3- to 9-fold) in the presence of moderate concentration of denaturants (20% (v/v), DMSO, 2.5 M urea or 1 M GdnHCl) and increased thermal stability under these conditions. All three enzymes in this study rM4, M6 and CyM6 can catalyse peptide ligation at 60 °C, in presence of 1 M GdnHCl, or 2.5 M urea unlike WT SaSrtA.

### 2.2 Peptidyl asparaginyl ligases

The second major class of peptide ligases, peptidyl asparaginyl ligases (PALs) (Scheme 2D), are closely related to asparaginyl endopeptidases (AEP). Like sortase these are cysteine proteases but have a significantly shorter recognition motif. Both PALs and AEPs bind a tripeptide recognition motif, 
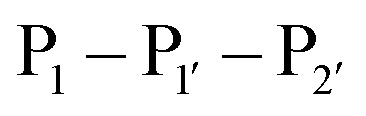
 (where P_1_ is asparagine or aspartic acid, 
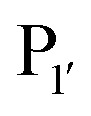
 is a small residue and 
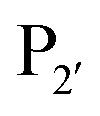
 is generally a hydrophobic/aliphatic amino acid *e.g.* NGL).^71^ In general AEPs act purely as proteases; under acidic conditions, AEPs hydrolyse the 
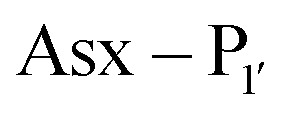
 bond.^71,72^ However, as the pH is increased, while AEPs lose the ability to bind aspartyl-containing substrates due to the loss of hydrogen-bonding to a key residue in the S_1_ pocket of the enzyme, the asparaginyl-containing substrates are not affected by a change in pH.^72–75^ At these higher pHs, amine nucleophiles can act as acyl acceptors and for some AEPs, a ligation reaction can occur with an asparaginyl-containing substrate, however the ratio of ligation to hydrolysis is dependent on the AEP and sequence of the substrate and, most AEPs are predominantly proteases and not synthetically useful.^73,74,76–82^

PALs, which are exclusively found in plants, are characterised by their ability to catalyse 
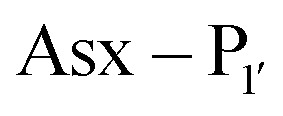
 bond formation in near-neutral conditions. These enzymes are best exemplified by Butelase-1,^76^ and OaAEP1^79^ whose endogenous activities are the production of cyclic peptides. PALs cleave the 
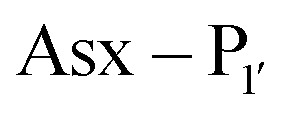
 bond to form a thioester intermediate which is then attacked by an N-terminal nucleophilic acceptor (X_1_X_2_) to form a new peptide bond with the P_1_ residue (Scheme 2D). The specificity for the N-terminal substrate is often even looser than the C-terminal tripeptide recognition motif allowing a wide variety of sequences in the product peptide. Hemu *et al.*^81^ proposed that the difference in activity between AEPs and PALs is due to the amino-acid composition of the substrate binding grooves flanking the S_1_ pocket of the enzymes, particularly the ‘gatekeeper’ residue (termed the ligase-activity determinant 1 region, LAD1) and residues found in LAD2 that are centred around the S_2_ and 
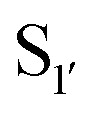
 pockets. Combining structural analysis and mutagenesis studies, it was determined that, for an efficient PAL, the first position in LAD1 is preferably bulky and aromatic (Trp/Tyr) and the second position (the gatekeeper) is hydrophobic (Val/Ile/Cys/Ala). Conversely, a Gly at the gatekeeper position favours proteolysis as is observed in the AEPs. For LAD2, small hydrophobic dipeptides (*e.g.*, GlyAla/AlaAla/AlaPro) are favoured in PALs as they retain the leaving group, blocking access to the thioester bond until another peptide acts as a nucleophile. In the case of AEPs, a bulky residue such as Tyr at the first position may destabilize the acyl–enzyme intermediate by facilitating the departure of the cleaved peptide group and exposing the acyl–enzyme thioester to water. Using this insights they were able to re-engineer a protease from *Viola candadensis* (VcAEP) into an effective peptide cyclase using a single point mutation of this Tyr residue to an alanine in the LAD2 region.

#### 2.2.1 Butelase-1

Butelase-1 was the first PAL to be identified and exploited. It was originally purified from seedpods of the plant *Clitoria ternatea* where it is involved in the biosynthesis of cyclotides.^76^ Butelase-1 natively catalyses head to tail cyclisation of peptides through recognition of a C-terminal Asx–His–Val motif (Scheme 2E).^83^ The enzyme cleaves the His–Val dipeptide and attaches the Asx residue to the N-terminal X_1_X_2_ sequence of the peptide, where X_1_ can be any amino acid, except Pro, and X_2_ is favoured to be a bulky hydrophobic amino acid such as Ile, Leu, Val and, to some extent, Cys. This leads to the cyclisation product with a new Asx–Xaa peptide bond. In this kind of application, butelase-1 has been utilised for the head-to-tail cyclisation of large circular bacteriocins.^84^ The enzyme can also be manipulated for use in ligation of proteins/peptides. For this reaction, peptide/protein 1 must carry the C-terminal Asx–His–Val recognition sequence and must not contain a Ile/Leu/Val/Cys residue at the X_2_ position at its N-terminus. On the other hand, peptide/protein 2 must contain the Ile/Leu/Val/Cys residue at the X_2_ position. This specificity means that butelase-1 can be readily used for protein modification.^85^ For example, a peptide containing a C-terminal NHV motif can be coupled with a protein with an N-terminal GI dipeptide. The ligation product will have a NGI sequence and not be a substrate for butelase-1. Conversely, for C-terminal protein modification, a NHV motif is required at the C-terminus of the protein which may need to be introduced into the protein. For example. Tam *et al.*^86^ carried out labelling of a HER2-specific DARPin (designed ankyrin repeat protein), having a C-terminal NHV motif, with a fluorescent peptide containing an N-terminal RIGK motif for bioimaging as well as to ligate cytolytic peptides to generate candidate therapeutic drug conjugates.

A distinct advantage of Butelase-1 is that it is the fastest known ligase with a very high catalytic efficiency. A typical butelase-mediated reaction requires 100- to 1000-fold less enzyme than a reaction carried out with sortase A. It also has a shorter recognition sequence than sortase (Asx–His–Val) and broader tolerance for the first N-terminal residue for intermolecular peptide and protein ligations, however it is limited by the identity of the second residue. The first applications of butelase-1 were chiefly limited by its availability, since it could only be obtained by extraction from plant tissue. Nguyen *et al.*^76^ first attempted to recombinantly express the enzyme in *E. coli* in 2014, however it was only expressed in an insoluble form. Only very recently have James *et al.*^87^ successfully expressed recombinant butelase-1 in *E. coli*. The enzyme was produced as an inactive zymogen, which is the native form of AEPs and PALs, and matured by autoactivation at low pH in a protocol mimicking the natural process in the plants. The recombinant protein possessed a His_6_ tag at its N-terminus followed by a GS linker and the fully encoded butelase-1 (minus the 20-residue endoplasmic reticulum signal peptide). After purification of the N-terminally His-tagged zymogen, dialysis at pH 4.0 led to cleavage of the C-terminal propeptide which blocks the active site as well as the N-terminal propeptide. As part of the same study, the crystal structure of the purified zymogen was solved which will potentially allow engineering of butelase-1 to avoid the need for an activation step in the future. In contrast to this multi-step protocol from *E. coli*, butelase-1 could be successfully produced following overexpression in the yeast *Pichia pastoris*.^88^ In this case, export into the ER of the yeast cells also enhanced the formation of disulfide bonds between the five cysteine residues present in butelase-1 enabling folding of the active enzyme. The availability of recombinant butelase-1 will open many more opportunities for protein engineering in the near future.

Due to the earlier lack of a recombinant expression system that limited supplies, most studies of butelase-1 activity have demonstrated its application following immobilisation. For example, Hemu *et al.*^89^ immobilised butelase-1 using three different attachment methods: non-covalent affinity capture using both concanavalin A-agarose beads that recognise butelase-1 glycans and NeutrAvidin beads binding to the biotinylated enzyme, as well as covalent attachment *via* direct coupling of amines to NHS ester-functionalised agarose beads. The immobilised butelase-1 was reusable for >100 runs with undiminished activity, lowering the consumption of enzyme. Immobilisation also enhanced the stability and prolonged the shelf life of the enzyme compared to the soluble form by reducing aggregation and autolysis into less active forms. In particular, the immobilisation increased the effective concentration of the enzyme, accelerating catalytic activity of ligation reactions such as cyclisation and cyclo-oligomerisation under one-pot conditions or in a continuous flow-reactor.

#### 2.2.2 OaAEP1

A second PAL, OaAEP1 from *Oldenlandia affinis*, which shares 66% sequence identity with Butelase-1 is also a catalytically efficient ligase that can be used for protein modification. It is reported to be 90 times slower than butelase-1, but has been fully characterised and was first recombinantly expressed in bacteria as a zymogen that required further processing at low pH to generate its active form.^79,90^ A single site-directed mutant (C247A) is sufficient to increase the activity of OaAEP1 160-fold^80^ and the catalytic domain of this mutant has been successfully produced as a His-tagged ubiquitin fusion protein by Tang *et al.* which does not require activation. The C247A mutation also relaxes the specificity at the 
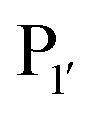
 position, which has been attributed to the presence of the smaller side chain, and OaAEP1 C247A can cleave the peptide bond between asparagine and all 20 amino acids except proline.^90^ The specificity at the 
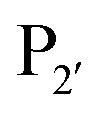
 position appears to be for large hydrophobic residues such as Phe, Ile, Leu, Met and Trp and it only poorly hydrolyses sequences containing Val (Scheme 2E).^90,91^ Residues G and L at 
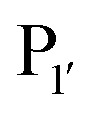
 and 
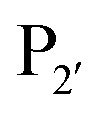
 are one of the most effective combinations. In this case, the enzyme recognises a C-terminal NGL motif, resulting in the formation of a protein–enzyme thioester intermediate. Nucleophilic attack with an N-terminal GL-containing substrate results in a NGL-containing ligation product.^80,91^ OaAEP1 C247A has been used for both N- and C-terminal site-specific protein modification.^91^ For example, OaAEP1 was used by Deng *et al.*^92^ to build protein polymers using head-to-tail protein–protein ligation and Harmand *et al.*^93^ used it to modify the surface of red blood cells with nanobodies.

#### 2.2.3 VyPAL2

VyPAL2, from *Viola yedoensis*, was recently identified as a third highly active PAL.^81^ The proenzyme was expressed in insect cells and autoactivated at acidic pH to yield the active enzyme. Substrate specificity studies, *via* cyclisation of peptides containing C-terminal 
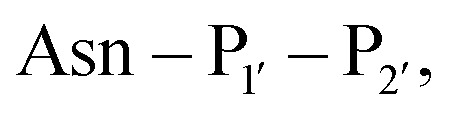
 revealed that small amino acids, particularly Gly and Ser, are favoured at 
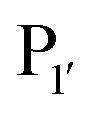
 but not Pro (Scheme 2E). The 
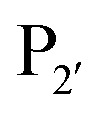
 position favours the presence of hydrophobic or aromatic residues, such as Leu/Ile/Phe. Kinetic studies showed cyclisation of a model peptide could be achieved with a catalytic efficiency of 274 000 M^−1^ s^−1^, only 3.5-fold less than butelase-1 (972 000 M^−1^ s^−1^). VyPAL2 has ligase activity at near-neutral pH and displays minimal hydrolase activity even at low pH, making it an attractive ligase for protein labelling. As described for butelase-1 above, VyPAL2 was also subject to immobilisation in the same study by Hemu *et al.*^89^ Immobilised VyPAL2 also showed increased activity, reusability and stability compared to its soluble counterpart. The use of this enzyme in ligation reactions is described in greater detail in Section 5.3.

### 2.3 Subtiligase, stabiligase and peptiligase

Subtiligase is an engineered ligase produced *via* modification of the serine protease subtilisin BPN’ from *Bacillus amyloliquefaciens via* two site-directed mutations (Scheme 2F).^94^ Mutation of Ser221 to cysteine from the catalytic triad to form thiolsubtilisin had previously been shown to enable catalysis of peptide formation from peptide ester substrates due to the formation of a thioester intermediate which is resistant to hydrolysis.^95^ Subtiligase was generated *via* a second P225A mutation which reduces the steric crowding in the active site (a result of the first mutation).^94^ This enzyme reacts two orders of magnitude faster with peptide ester substrates than thiolsubtilisin. Reaction of the thioacyl intermediate is selective for N-terminal α-amines over lysine ε-amines. A second subtilisin variant with the nucleophilic serine replaced by selenocysteine, termed selenolsubtilisin, has also been reported.^96^ While the selenoester intermediate formed means this is 20 times more efficient than thiolsubtilisin at catalysing aminolysis, it is much more susceptible to oxidative inactivation than subtiligase.

The substrate specificity of the acyl-donating side of subtiligase is assumed to be retained from subtilisin BPN′, which has been extensively studied structurally and biochemically.^97–105^ However, acyl acceptor preference screening has been carried out specifically for subtiligase.^106,107^ In particular, an approach called proteomic identification of ligation sites (PILS) has been applied for identifying N-terminal substrate specificity.^107^ Using peptides derived from proteolysis of *E. coli* cell lysates it is possible to rapidly profile the ligation efficiency for >25 000 different potential substrates which can then be identified by isolation and sequencing of ligated peptides *via* LC-MS/MS. This allowed rapid determination of the preferred substrate specificity (Scheme 2G). The 
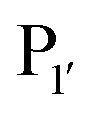
 position preferentially binds small amino acids, Met or basic residues, and the 
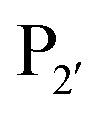
 position is preferentially aromatic, large, and hydrophobic. Mutation of subtiligase was also used to map residues in the enzyme which lead to this specificity revealing that Tyr217 and Phe189 are the primary determinants of 
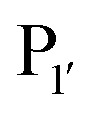
 and 
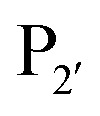
 specificity, respectively.

Subtiligase has been utilised in many applications including peptide cyclisation,^106^ the synthesis of thioesters^108^ and the synthesis/semi-synthesis of large proteins.^109^ For example, Wells *et al.*^109^ used the enzyme to perform total synthesis of Ribonuclease A from six peptide fragments. Due to the chemo-selectivity of subtiligase for the protein N-terminus, the enzyme can be utilised for site-specific protein modification.^106^ The first example of this was the modification of human growth hormone where the N-terminal structural and sequence requirements for efficient ligation were explored. In this case, it was discovered that introducing an extended N-terminal sequence to the protein resulted in higher modification yields as is often the case for other peptide ligases. Other advantages of the enzyme are that it can be recombinantly expressed in high yields and only requires a sub-stoichiometric amount of enzyme. The principle disadvantages of subtiligase are, however, that the enzyme only works on peptide ester substrates as acyl donors and that a large excess of acyl acceptor/donor is required to suppress the hydrolytic reaction. Near quantitative ligation of peptide substrates could be obtained using a 10-fold excess of some acyl acceptors, suggesting that this approach had promise for peptide assembly but that further optimisation was required.^94^

#### 2.3.1 Increased stability

Several different reports of engineering to enhance the behaviour of subtiligase have been published. In some cases, the stability of the catalyst was thought to limit its activity on certain substrates.^106^ Five stabilising mutations (M50F, N76D, N109S, K213R, and N218S), previously identified in subtilisin to enhance stability to heat, basic conditions and organic solvents were introduced into subtiligase. This new variant, termed stabiligase, is capable of activity under the conditions required to label proteins previously shown to be resistant to subtiligase modification, thus expanding the applicability of subtiligase-mediated protein modification. The improvement in activity of subtiligase as a result of the P225A mutation^94^ has inspired further mutation studies to enhance activities. *In vitro* screening of a large library of subtiligase mutants, each with four to five mutations near the active site, led to the identification of two new double mutants (M124L/S125A and M124L/L126V) with ligation rates greater than two-fold improved compared to the original subtiligase.^110^ Many of the other identified variants contained conserved residues known to improve the thermodynamic or oxidative stability of subtilisin *e.g.* N218S dramatically stabilises subtilisin to heat denaturation. The *in vitro* screening approach also showed that the original P225A mutation was largely preserved in highly active mutants with glycine being the only other residue tolerated at this position.

Both subtilisin and subtiligase are calcium-dependent due to the presence of a calcium-binding domain required for efficient folding of the proteolytic domain. Deletion of this domain from subtiligase and addition of a set of 18 stabilising mutations previously identified for subtilisin^111^ yielded a calcium-independent variant of subtilisin, peptiligase. This enzyme can be easily expressed in *Bacillus subtilis* and has high catalytic efficiency.^112^ Peptiligase catalyses peptide bond formation between C-terminal carboxamidomethylester fragments and N-terminal acyl-acceptor nucleophiles. In this case, the peptiligase-mediated reaction is very selective for peptide ligation over the hydrolysis given conversions of 60–80% using only 1.5 equivalents of acyl acceptor. Peptiligase was also used to synthesise head-to-tail macrocyclic peptides, producing a 21-mer macrocycle with a yield of 82%. The enzyme was also shown to be functional in the presence of organic solvents and denaturants. Synthetic peptide libraries have subsequently been used to map the specificity of the acyl-acceptor side of peptiligase.^113^ Unlike subtiligase, peptiligase accepts only the small amino acids Ser, Gly and Ala at the 
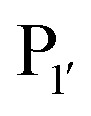
 position, dictated due to interactions with Met213 and Leu208 in the enzyme (analogous to Met222 and Tyr217 in subtiligase) while a hydrophobic residue is still required at the 
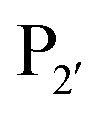
 position. While effective for peptide couplings, the overall substrate concentrations typically used (10 mM) in reports of subtiligase-mediated reactions are typically at least an order of magnitude higher than would typically be used for protein modification reactions and the majority of reports of this peptide ligase have been in peptide rather than protein applications as discussed later.

## 3 Substrate engineering to enhance product yield

As well as engineering of the enzymes to improve the efficiency of ligation reactions, efforts have also been made to engineer the substrates of the enzymes. This work has generally been focused on addressing the reversibility of the enzymatic reactions since ligation reactions otherwise often require a large excess of nucleophilic substrate and catalyst to push the equilibrium towards the formation of the desired ligation product.

### 3.1 Sortase A

**Scheme 4:**
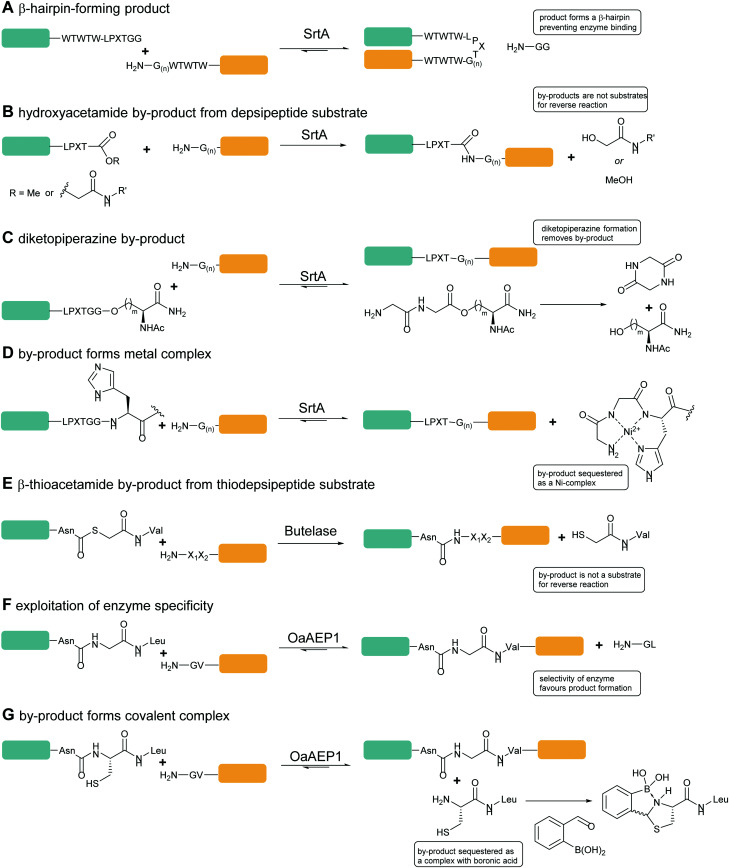
Substrate engineering strategies employed to enhance product yields with SrtA, Butelase and OaAEP1. (A) Formation of a β-hairpin prevents binding of SrtA to the reaction product.^115^ (B) Hydroxyacetamide products are not substrates for the reverse reaction.^25^ (C) Cyclisation of the diglycyl motif with loss of serine generates a diketopiperazine.^117^ (D) A GlyGlyHis motif is a ligand for Ni_2+_ in solution which sequesters the product peptide as an inactive complex.^118^ (E) β-Thioacetamide products are not substrates for the reverse reaction.^119^ (F) Enzyme selectivity is exploited: while OaAEP1 can act on a NGL sequence to form an NGV product, the NGV sequence is a poor substrate.^91^ (G) The product peptide with an N-terminal cysteine is sequestered by formation of a complex.^90^

Sortase-catalysed reactions between peptide and protein substrates are reversible since the products of the ligation reaction are also substrates for sortase. Reaction of sortase with an LPETGX motif in a substrate to form a thioester intermediate generates a GlyXaa dipeptide. This then competes with the acyl acceptor substrate for the acyl–enzyme intermediate. Similarly, this acyl–enzyme can be re-formed from the desired ligation product and so the reaction can be effectively reversed and will eventually just go to equilibrium depending on the relative concentrations of species.^25,114^ A large concentration of one, or other, component of a labelling reaction can be usedto drive the reaction towards completion. Several distinct approaches to reduce the need for excess reagents have been taken. For example, Yamamura *et al.*^115^ used secondary structural elements to prevent the reverse reaction by generating an unreactive β-hairpin at the LPXTG ligation site in the product (Scheme 4A). Sortase-ligation between substrates containing WTWTW-LPXTGG and GG-WTWTW motifs produced a product with a stable secondary structure element that inhibited recognition of the product LPXTG motif by sortase. Although successful, this technique involves introduction of a relatively large additional peptide sequence with a secondary structure which could disrupt protein function.

A second, more widely adopted approach is to use substrates which generate an inactive by-product. An example of this is using ester-containing substrates to modify a protein which lead to an alcohol-containing by-product which is not a substrate for the enzyme thereby rendering the reaction irreversible (Scheme 4B). Antos *et al.*^114^ first demonstrated this with methyl ester containing substrates (Scheme 4B; LPRT-OMe), however stoichiometric quantities of sortase and excess substrate was required to achieve quantitative labelling presumably since the methyl ester was a poorer substrate for sortase than the peptide product. Williamson *et al.*^25^ instead generated depsipeptide substrates which more closely mimicked the peptide substrate in that only the amide nitrogen of the Thr-Gly linkage was replaced with an oxygen to generate an ester linkage (LPEToGG). This technique was used to label a range of proteins with essentially quantitative ligation yields using around 2–3 equivalents of the labelling reagent and 20 mol% sortase.^25,116^ Williamson's results showed that depsipeptide substrates allow rapid labelling of both peptides and proteins using a small excess of substrate and catalytic quantities of sortase. An alternative ester substrate generated by Liu *et al*.^117^ placed the ester linkage outside the sortase recognition motif.

In this case, LPETGG-isoacyl-Ser/Hse containing substrates were used to N-terminally modify a protein (Scheme 4C). Upon ligation, the released by-product spontaneously cyclises to generate diketopiperazine. One potential advantage of these substrates is that these esters are reportedly more stable than Antos and Williamson's substrates. Despite these disadvantages, depsipeptide substrates have seen numerous applications including in applications such as profiling N-terminal glycine containing proteins.^120^ Most recently this approach has been used by Wang *et al.* in combination with a HPXTG-specific sortase to generate a wide range of engineered histone H2B variants with complexly modified N-termini.^121^

In a third approach, Row *et al.*^118^ employed a technique that deactivated the by-product through nickel-coordination. (Scheme 4D) In this case, the labelling substrate motif was extended to LPXTGGH; the GGH tripeptide formed as a result of reaction chelates Ni^2+^, thereby sequestering the product and inhibiting participation in the reverse reaction. Building on this work,^122^ the group worked to further develop and optimise this metal-associated sortase-mediated ligation (MA-SML) approach through peptide model studies to establish the structural features of ligation substrates and nucleophiles. With the extended C-terminal recognition motif, LPXTGGHH_5_, and a solution additive (Ni^2+^), modification of full-size proteins with fluorophores, PEG and a biorthogonal cyclooctyne moiety was achieved. An advantage to the MA-SML approach is that it can be applied to both N-terminal and C-terminal sortase labelling, unlike the ester approach which is only appropriate for N-terminal labelling. However large quantities of Ni^2+^ are required for this approach and this may not be compatible with all protein systems or for *in vivo* application.

In all of the approaches described above, the general strategy is to in some way chemically ‘remove’ the by-product species from the reaction equilibrium in order to drive the reaction to completion. An alternative approach, pioneered by Freiburger *et al.*^123^ for the preparation of segmentally labelled samples for NMR is to physically remove the by-product from the reaction mixture. This removal can be achieved by carrying out coupling reactions in centrifugal concentrators, such that the product peptide (which is smaller than the molecular weight cutoff of the device) is removed from the reaction mixture by cycles of concentration and dilution. This approach can be effective where a C-terminal labelling species is large relative to the peptide product and where the proteins involved can tolerate repeated cycles of centrifugal concentration.

Cong *et al.* have recently described a different approach towards the engineering of sortase substrates.^124^ They focused on the limitations of producing proteins with N-terminal glycines for N-terminal labelling which did not rely on the action of methionine aminopeptidase or signal peptidase in the cell or the use of engineered recognition sites for proteases such as TEV protease to reveal the N-terminal glycine sequence. To address this challenge, Cong *et al.* developed a one-step ‘swapping’ approach for the site-specific N-terminal sortase-labelling/protein-fusion of recombinantly produced proteins. Proteins were overexpressed including an N-terminal MH_6_-LPETG_5_-motif, addition of sortase then revealed the glycine motif *in situ* enabling coupling to a labelling peptide which also contained the sortase motif. While this approach worked well for the near-quantitative labelling of the protein, a substantial excess (5–15-fold) of the labelling peptide was required. This approach was also used to produce C–N protein fusion VHH-GFP *via* the sortase-mediated coupling of VHH-LPETGGH_6_ and MH_6_LPETG_5_-GFP, in this case, while product was formed an excess of the VHH-LPETGGH6 protein was required to drive ligation.

### 3.2 Butelase-1

Just as for sortase, peptide ligations with butelase-1 require an excessive amount of substrate (>5-fold) to compete with the cleaved dipeptide, His–Val, which acts as a competitive nucleophile to reverse the ligation reaction. Inspired by the use of depsipeptides in combination with sortase, Nguyen employed a similar technique for butelase-mediated conjugation.^119^ The group used thiodepsipeptide substrates for conjugation reactions (Scheme 4E). Quantitative ligation yields of >95% for a model peptide at 0.0005 molar equivalents of butelase 1 and two molar equivalents of thiodepsipeptide were achieved. The technique was also used to site-specifically modify the N-terminus of ubiquitin and green fluorescent protein in high yields. Again as for sortases, the downside to using thiodepsipeptides in this manner is their short half-lives and the technique is limited to N-terminal labelling.

### 3.3 OaAEP1

OaAEP1-mediated conjugation is also subject to reaction reversibility. This problem was addressed by Rehm *et al.*^91^ who, rather than focusing on deactivating the side product, explored the enzymes tolerance for alternative nucleophiles (Scheme 4F). A GV-containing nucleophilic peptide was shown to achieve efficient ligation comparable to that of the GL-containing peptide. However, the NGV-containing ligation product was poorly cleaved compared to the NGL-containing product, being processed by OaAEP C247A with a 50-fold lower specificity constant (*k*_cat_/*K*_M_). Subsequently, nanobodies with a C-terminal NGL or NGV extension were generated. The NGL-modified nanobody was efficiently labelling with a GV-based fluorescent peptide to yield an NGV-containing ligation product with 90% conversion, whereas the NGV-modified nanobody achieved less than 2% ligation product. The same approach could also be used for cyclisation. eGFPs with C-terminal NGL motifs and N-terminal GL or GV motifs were rapidly cyclised to 90% completion, but GV-eGFP-NGV was resistant to cyclisation. Finally, they demonstrated labelling on a nanobody construct, achieving 80% labelling of either an N-terminal GVG motif using NGL-terminated probes or a C-terminal motif using GV-terminated probes. Notably, while less catalyst was required for N-terminal labelling, a greater number of equivalents of the probe were required to get equivalent labelling. In both cases, the inertness of the NGV motif formed as a result of the reaction is key to favouring product formation.

Iwai and co-workers showed that the OaAEP1 C247A variant also recognises a NCL motif.^125,126^ This property was utilised by Tang *et al.*^90^ to develop an alternative chemo-enzymatic strategy to reduce the reversibility of the OaAEP1-mediated ligation reaction for both N- and C-terminal labelling. In their approach, the CL-terminated peptide, formed as a result of ligation between a C-terminal NCL motif and an N-terminal GL is sequestered *via* reaction with 2-formylphenylboronic acid to form a thiazolidine (Scheme 4G).^127,128^ The reaction is also extremely efficient, with a bimolecular rate constant of up to 10^5^ M^−1^ s^−1^. The technique was utilised for both site-specific C-terminal and N-terminal protein labelling.^90^ Using 2 equivalents of a labelling peptide it was possible to achieve between 75% and 92% labelling on C-termini and 79% on the N-terminus. The high yields achieved with only 2 eq. of labelling substrate illustrates the effectiveness of the approach at a relatively low label-to-protein ratio.

### 3.4 Subtiligase

Due to the nature of how subtiligase was developed, an ester linkage is required at the C-terminus of the donor substrate for ligation reactions. In general lactate-derived substrates are preferred to the equivalent glycolate substrates with values of *K*_m_ 5–10-fold lower but in both cases further extension of the substrate with amino-acid residues enhances binding.^94^ Tan *et al.*^129^ demonstrated the superiority of peptide thioester substrates over peptide esters. Using model acyl donors, a thioester substrate was shown to achieve quantitative ligation in just 3 min, compared to a peptide ester substrate that took 65 min due to a 10–20-fold increase in *k*_cat_. The requirement for peptide ester and thioester substrates largely limits their application to N-terminal protein labelling, however thioester substrates for C-terminal labelling have been generated by use of a modified intein which allows formation of a C-terminal benzyl thioester.^130^ This approach enables recombinant expression of C-terminally thioesterified proteins and subsequent labelling using subtiligase.

## 4 Enzyme engineering to broaden substrate scope

One of the great advantages of using enzymes for peptide ligations is that they are inherently sequence specific. However, sometimes the strict substrate specificity can be a hindrance, limiting the applications of the technique. Thus, efforts have been made to engineer the enzymes to broaden the substrate scope.

### 4.1 Sortase

The wild type sortase from *S. aureus* (SaSrtA) only accepts substrates containing an LPXTG sequence. This constraint prevents the use of these enzymes to modify endogenous proteins that lack this sequence.^64^ The range of available sortases has been broadened both by exploitation of sortases from other species and re-engineering of SaSrtA.

#### 4.1.1 Sortases with alternative substrate specificity

Although sortase A is the principal class of sortase to have been exploited, multiple distinct classes of sortases are found in bacteria.^11–13^ Beyond SaSrtA, Das *et al.*^13^ investigated Sortase E from *Streptomyces avermitilis* (SavSrtE) which was known to perform a housekeeping role in its host. The enzyme prefers a LAXTG recognition motif over LPXTG and is naturally Ca^2+^-independent, presenting SavSrtE as a candidate for development into a tool for protein conjugation to complement SaSrtA. Other accessory sortases such as sortase B from oral streptococci^131^ and sortase D from *B. cereus*^132^ are generally involved in linking particular proteins to the cell surface or in pili assembly. For example sortase D is able to catalyse the linkage of an IPNTG derived acyl donor in the protein BcpB to internal lysine residues in YPKN motif at the tip of bacterial pili. In general however these proteins have not been extensively exploited.

As discussed earlier, SpSrtA from *S. pyogenes*, which is also Ca^2+^-independent, has been used in protein labelling reactions, accepting both LPXTG and LPXTA motifs.^114^ Nikghalb *et al.*^133^ have subsequently investigated the substrate specificity of a range of sortase A enzymes of staphylococcal and streptococcal origin. In general, streptococcal sortases accept a broader range of substrates then SaSrtA, including LPXTG, LPXTA and LPXTS motifs and consequently N-terminal Gly, Ala and Ser nucleophiles. In particular, *Streptococcus pneumoniae* sortase A, that recognises the LPXTS substrate, was used for site-specific modification of the N-terminal serine residue of a 48-residue antimicrobial peptide. Additionally, Schmohl *et al.*^134^ determined that streptococcal sortases show a strong preference for an LPXLG motif over LPXTG. These results highlight the potential for alternative sortases but many of these have not been extensively exploited, often due to the low catalytic activity of the isolated enzymes.

Zou *et al.*^66^ have recently reported the design of SpSrtA variants with improved transpeptidase activity towards different N-terminal amino acid residues. Based on sequence alignment of sortase A from different species they identified conserved residues near the active site suitable for mutation. Three SpSrtA variants (S141G, V206I, and T209D) were generated and assayed using a protein fusion system between a C-terminal LPETG motif and an N-terminal AA-motif. SpSrtA V206I showed significantly improved activity in comparison to WT SpSrtA. Subsequently, site-saturation mutagenesis in the β6/β7 and β7/β8 loops using the optimised SortEvolve^47^ high-throughput assay described above led to identification of four variants (E189H, E189V, E215A and E215G) with improved activity (≥1.3-fold). The SpSrtA E189H/V206I/E215A M3 triple mutant showed 6.8-fold increased transpeptidase activity when compared to WT. This catalyst could then be used for conjugation of AA-, SS- and CC-terminated motifs to model proteins and for circularisation of eGFP constructs with N-terminal AA and SS-motifs.

The sortase from *Corynebacterium diphtheriae* (CdSrtA) (pilus-specific enzyme), has also been exploited for protein modification.^61,135^ The enzyme was originally considered to be a sortase A enzyme, thus named accordingly. However, unlike sortase A, CdSrtA functions as a pilin polymerase and therefore can be categorised into the C family. The enzyme covalently links SpaA pilin subunits together *via* lysine-isopeptide bonds. This linkage is between an internal WxxxVxVYP**K** pilin motif in the N-terminal domain and a C-terminal LPLTG motif. Following formation of an acyl–enzyme intermediate between catalytic Cys222 and the LPLTG motif, the intermediate is then attacked by the reactive Lys190 residue within NSpaA's pilin motif resulting in a Thr494–Lys190 isopeptide bond between CSpaA and NSpaA domains within adjacent pilin subunits. The overexpressed WT CdSrtA is catalytically inactive *in vitro* due to the presence of an N-terminal polypeptide lid segment that masks the enzyme's active site. Introduction of D81G and W83G lid mutations activates the enzyme and a soluble catalytic domain with these mutations is able to site-specifically ligate the isolated NSpaA and CSpA domains *in vitro*.^135^ Introduction of a third mutation (N85A) further increases activity leading to 35% more product after a 24 h incubation. The conjugation reaction catalysed by CdSrtA 3M enables site-specific lysine labelling, creating an isopeptide bond but requires two specific motifs and is currently limited in yield, nonetheless it does provide an interesting avenue for future engineering studies.

#### 4.1.2 Engineering the specificity of sortase A

The sortase A from *Staphylococcus aureus* (SaSrtA) has been extensively engineered to accept different recognition sequences. Such engineering was first attempted by Bentley *et al.*^62^ who recognised that, although the accessory sortase SaSrtB is analogous to SaSrtA, it has a different specificity profile and is highly specific to the NPXTN-containing IsdC protein *in vivo*.^58^ Despite SaSrtA and SaSrtB having differing β6/β7 loop compositions, they occupy equivalent structural positions and likely both function as the main contact site between sortase and the recognition motif. By this logic, a loop swap chimera, SrtLS_ΔN24_, wherein the β6/β7 loop of SrtA was exchanged with the corresponding SrtB loop, was generated.

This chimeric protein consisted of the SrtB Lys174–Asp215 loop inserted between SrtA Asp160 and Lys177 (renumbered to Lys203 as the SrtB loop is 26 residues longer). This replacement of the β6/β7 loop in SrtLS was sufficient to change the specificity profile for NPQTN by over 700 000-fold, verifying that the β6/β7 loop is the primary substrate recognition site. However, SrtLS was only able to catalyse the hydrolysis of the motif and not the ligation reaction. This may indicate that the swapped loop could prevent the nucleophilic substrate accessing the active site or there may be additional domains in the SrtB enzymes that are necessary for transpeptidation. Nevertheless, the study illustrated that engineering the substrate specificity of SaSrtA enzymes has potential.

In an alternate approach, Piotukh *et al.*^45^ demonstrated the first use of directed evolution to identify a SrtA mutant that possesses broader substrate specificity. To achieve this, a library of 10^8^ sortase mutants was constructed, designed to screen for sortases that recognise the FPXTG motif. This motif was selected as bioinformatics approaches indicate that it exists in nature and marginal ligation of this motif has also been observed using SaSrtA. To produce the library, six amino acids in the β6–β7 loop of sortase, representing solvent-exposed positions in spatial proximity to leucine in the LPXTG motif, were randomised. The library of sortases were generated with N-terminal pentaglycine motifs and C-terminally fused to the pIII protein of M13 phages (Fig. 1B). Exposure of the phage library to biotin-GFPKTGGRR-NH_2_ peptides therefore led to covalent modification of those phage with mutations that promoted the ligation reaction but not hydrolysis, phage encoding active mutants could then be accumulated *via* streptavidin capture. Three rounds of selection yielded a set of four mutants that, following subcloning and overexpression, were shown to tolerate a range of amino acids at the first position in the motif. Of these, the F40 mutant (Table 1) was identified to prefer FPXTG to LPXTG, but ligation efficiency was low. However, the mutant had remarkably broad specificity, and actually had a preference for Ala in the first position of the motif. Ligation reactions using an APKTG-containing peptide with SrtA-F40 resulted in 55% labelling after 24 h. Although the mutant has reduced activity compared to SaSrtA, it could still be used to modify histone H3, a protein that has a native APATG motif located at the interface between the globular fold and the tail.

Building upon this work, Schmohl *et al.*^63^ established a second generation sortase library, with the β6–β7 loop randomised at nine positions, based on a more recently determined NMR structure of sortase A.^136^ This new structure had revealed a different conformation for the β6–β7 loop in the bound substrate state which indicated that the initial residues selected for randomisation may not have been ideal for evolving SaSrtA. Thus, a redesigned SaSrtA library was generated, including variation of β6–β7 loop length. The library comprised of approximately 2 × 10^8^ mutants and was screened *via* the previously established phage display system to identify mutants that accepted substrates containing APXTG or FPXTG recognition motifs. This led to the identification of the F1-21 mutant (Table 1) which accepted both sorting motifs efficiently and showed the highest activity of all sortase mutants isolated so far by phage display. The majority of the isolated mutants contained β6–β7 loops that were longer than the native loop.

In another study, Dorr *et al.*^64^ evolved two orthogonal sortase variants with altered specificity based on eSrtA, eSrtA(2A-9) with 11 mutations which recognises LAXTG and eSrtA(4S-9) with 9 mutations which recognises LPXSG (Table 1). The yeast display screen that had been used to evolve eSrtA was modified for this application, with the addition of a negative selection against recognition of off-target substrates.^44^ Nine rounds of yeast display screening with concomitant refinement of library design and screening strategy led to the evolution of variants of eSrtA that were reprogrammed to recognise new substrates with specificity changes of up to 51 000-fold relative to eSrtA and minimal loss of catalytic activity. Both eSrtA(2A-9) and eSrtA(4S-9) strongly prefer the LAXTG and LPXSG substrates, respectively, over the LPXTG substrate, with up to 24-fold specificity for their target substrates. Mutational dissection of the two variants revealed the importance of residue 104 for enzyme activity and specificity at position 2 of the sortase motif. In combination, residues 104, 118 and 182 determine the activity and specificity at position 4 of the sortase motif. Furthermore, eSrtA(4S-9) was demonstrated to modify human protein fetuin A (recognition sequence LPPAG) in unmodified human plasma with high efficiency and specificity, which was unachievable with WT or eSrtA. Both variants could be used to mediate rapid synthesis of double modified fluorophore-protein-PEG conjugates and to functionalise GGG-linked surfaces simultaneously and orthogonally with target peptides.

Recently, the substrate specificity of SaSrtA has been reprogrammed to modify the Alzheimer's disease (AD)-associated Aβ protein, which contains an LMVGG sequence at residues 34–38.^65^ The yeast display and FACs strategy used to evolve the eSrtA enzymes was also applied here.^64^ Evolution was started from one of the library of sortase variants previously evolved to recognise LPESG variants (4S.6).^64^ The rationale for this was that mutants already possessing altered substrate recognition at the fourth position would be a more promising start. After 16 rounds of evolution, involving diversification of the library pools for each round *via* error-prone PCR, site-saturated mutagenesis and DNA shuffling, SrtAβ was generated. This involved the decrease in concentration of biotinylated LPVGG as a positive selection substrate and decrease in off-target non-biotinylated LPESG. This was to increase the stringency of the screen as the rounds went on. The resultant Srt-Aβ enzyme had 25 amino acid changes (Table 1) compared to the parent sequence 4S.6. These mutations ranged from mutations at positions known to mediate sortase specificity to mutations at highly conserved residues in naturally occurring sortases. These diverse changes provide insights into mechanisms of sortase functions. Compared to the starting enzyme 4S.6, SrtAβ had a 53-fold reduced activity on LPESG, 11-fold reduced activity on LPPAG and 28-fold increased activity on LMVGG. Overall, the directed evolution process lead to a 1500-fold change in the preference of SrtAβ for LMVGG over LPESG compared to SrtA(4S.6) The evolved enzyme, SrtAβ, was used to generate conjugates with Aβ monomers using peptides such GGGK(biotin) and GGGRR, validating the evolution of epitope-specific enzymes as a strategy for site-specific labelling of endogenous peptides. SrtAβ could also conjugate peptides to endogenous Aβ in human CSF and is a promising tool for the study of amyloid proteins.

Piper *et al.*^137^ have reported the effect of mutation in the β7–β8 loop region on the activity of SpSrtA. As discussed above, this enzyme is able to act on an LPX_1_TX_2_ sequence where X_2_ is Ala, Ser or Gly but the activity is otherwise relatively low. Wojcik *et al.* had previously shown that grafting the β7–β8 loop from SaSrtA into SpSrtA generated an LPXTG specific enzyme^138^ however Piper *et al.* investigated the effect of creating SpSrtA chimeras where the β7–β8 loop from SrtA from a variety of other Gram-positive bacteria was grafted into the SpSrtA backbone.^137^ Many of these chimeras such as SpSrtA_*faecilis*_ (in which three amino acid) substitutions were able to catalyse reaction of LPX_1_TX_2_ peptide substrates faster than SpSrtA. Most interestingly, some of these enzymes were also able to act on a wider range of amino-acid nucleophiles including SpSrtA_*faecilis*_ which was shown to act on a LPXTV sortase recognition sequence.

Finally, most recently, Wang *et al.* used site-directed mutagenesis to combine mutations found in F40-Sortase (which has relaxed specificity for the first position in the recognition motif), eSrtA and the Ca-independent Srt7M and Srt7Y to generate a range of candidate sortases to act on a HPDTG motif found in histones.^121^ Screening against a fluorescent substrate peptide candidate containing this motif was sufficient to identify a mutant with suitable activity for use in subsequent generation of site-specifically modified histones.

### 4.2 Subtiligase

Subtiligase has also been the focus of efforts to broaden its substrate scope to diversify its application. Unlike sortase and butelase-1, the site specificity of subtiligase is not dependent on a specific recognition sequence. It relies on the ability of the enzyme to recognise an N-terminal α-amine. This broad substrate specificity towards α-amine peptide/proteins is advantageous as it allows sequence flexibility and leads to traceless ligation. However, it does have some restrictions regarding the acyl donor substrates it accepts which can be somewhat limiting. Protein engineering of subtilisin, focused on altering substrate specificity on the acyl-donor side, has proven to be translatable to subtiligase. Subtilisin favours hydrophobic or lysine residues at the P_1_ position. Through mutational studies, introduction of G166E, E156Q/G166K, and G166I mutations were identified to alter substrate specificity toward P_1_ Lys or Arg, Glu, and Ala, respectively.^94^ The same effects can be seen in subtiligase variants with the same mutations, allowing them to recognise specific P_1_ residues in the donor ester substrate.

In terms of the acyl-donor side, the mapping of the 
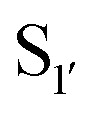
 and 
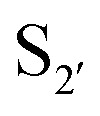
 pockets led to the production of a subtiligase mutant with altered substrate specificity for 
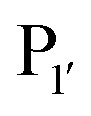
 and 
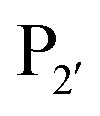
 residues.^107^ The mapping was achieved *via* alanine scanning and quantifying the resultant changes in ligation specificity using the PILS method. Based on the results, ‘hot spot’ positions 189 (
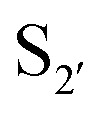
 pocket) and 217 (
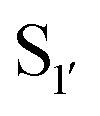
 pocket) were then targeted for saturation mutagenesis and the specificity profiles of the mutants were analysed using PILS. From this it was determined that Y217K/R mutants improved the reactivity towards sequences with an acidic 
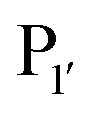
 residue, whereas Y217D/E mutants more efficiently modified a His, Lys, Ser or Arg 
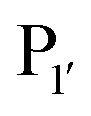
 residue. The F189Q/K/R mutants improved modification of peptides with an acidic 
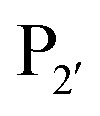
 residue. However, several F189 variants were expressed at much lower levels than WT subtiligase. Oxidation of Met222 is known to affect enzyme activity,^139^ and it also occurred in the enzyme variants. Mutation at the 222 position to alanine or glycine can improve subtilisin activity and enhance aminolysis to hydrolysis ratio in subtiligase.^113,140^ Thus, the F189 and Y217 mutations, along with M222A, were also introduced into the subtiligase heptamutant, stabiligase.^107^ The resultant variants were expressed at levels comparable to WT subtiligase and maintained the specificity profiles of the mutants. The introduction of the M222A mutation eliminated the methionine oxidation and improved the ligation to hydrolysis ratio.

To demonstrate application of these mutants,^107^ recombinant antibodies with N-terminal Ser-Asp on the light chain and N-terminal Glu-Ile on the heavy chain were produced. Based on the PILS specificity maps, these N-termini were predicted to be poor substrates for wild type subtiligase, and this was confirmed experimentally. The Y217K mutant quantitatively labelled the heavy chain, however, no measurable labelling of the light chain was observed using the F189R/M222A mutant. This was attributed to inaccessibility of the N-terminus and after addition of a four amino-acid linker, 62% ligation was achieved. To enable wider application of the generated mutants, a web-based tool, α-Amine Ligation Profiling Informing N-terminal Modification Enzyme Selection (ALPINE), was established to aid the selection of optimal subtiligase variants for modification of a particular N-terminal sequence.^107^ There has yet to be a mutant discovered that recognises all N-terminal sequences, thus selection of an appropriate mutant is important.

### 4.3 Peptiligase

Nuijens *et al.*^113^ focused on engineering peptiligase to improve ligation efficiency and broaden the substrate scope of the enzyme. Using structure-inspired protein engineering, the substrate profile of the 
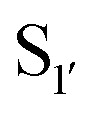
 pocket was radically broadened. As substrate scope of the 
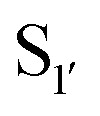
 pocket is largely controlled by Met213 and Lys208, replacement of Met213 with Ala, Gly or Pro and Lys208 with Gly, Ala, Ser or Asn broadened the tolerance of different 
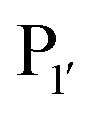
 residues. For peptiligases to favour intermolecular ligation over macrocyclisations, an N-terminal protecting group is required. However, in addition to peptiligase variants with broad specificity, engineering also yielded several variants with redesigned substrate profiles that allow selective peptide couplings without the need for N-terminal protecting groups. A selective peptiligase mutant was employed for the gram-scale synthesis of a pharmaceutical exenatide *via* multiple fraction condensations.

Omniligase-1, one of the broad specificity variants, is commercially available and has been used for chemo-enzymatic peptide synthesis (CEPS) of peptides,^142^ protein semi-synthesis and head-to-tail macrocyclizations of various linear peptides having a free N-terminus.^143^ Omniligase-1 provides an efficient inter- and intramolecular peptide ligation method for almost any peptide sequence and is scalable and robust enough for industrial application. For example, the enzyme was used in the large-scale synthesis of a 39-mer pharmaceutical exenatide.^144^

## 5 Orthogonal activity of peptide ligases

Exploration into broadening substrate scope of enzymes have allowed new opportunities to further expand the field of protein modification. An area of interest is to combine the use of orthogonal sortases with different substrate specificities to conjugate multiple substrates site-specifically onto a protein. This opens up a variety of opportunities to modify multiple sites on the same protein with a range of substrates leading to applications such as synthesis of biopharmaceuticals (*e.g.*, antibody–drug conjugates and vaccines) and probing of protein function and mechanism.

### 5.1 Sortase A

**Scheme 5:**
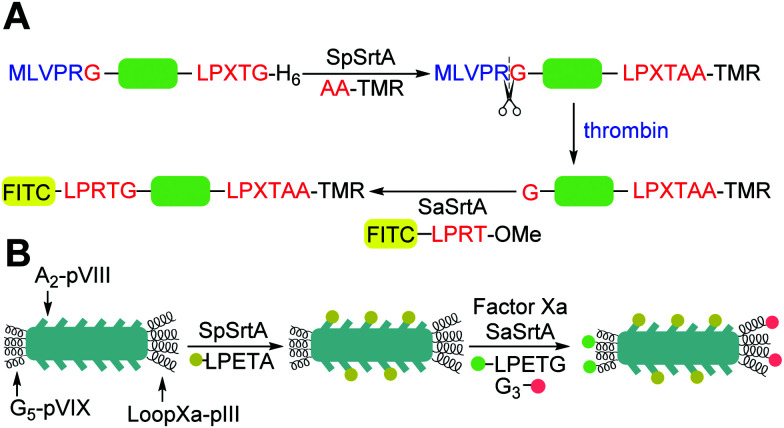
Examples of application of (A) strategy for the dual labelling of both termini of the same protein using SpSrtA and SaSrtA. Adapted from Antos *et al.*^114^ (B) Strategy for the triple labelling of distinct capsid proteins in a M13 bacteriophage particle. Adapted from Hess *et al.*^141^

Antos *et al.*^114^ first demonstrated orthogonal application of sortase A enzymes derived from two different species. The group developed a technique to site-specifically label the N- and C- terminus of a human UCHL3 protein using SpSrtA and SaSrtA (Scheme 5A). SpSrtA recognises both the LPXTA and LPXTG sequences, whereas SaSrtA is only specific for LPXTG. The C-terminus of UCHL3 was modified with an LPXTG sequence and a thrombin cleavage site was incorporated at the N-terminus. SpSrtA was used to ligate a rhodamine-conjugated peptide carrying an N-terminal alanine to the C-terminus of UCHL3. This produced a modified protein containing an LPXTA sequence. The protein was then treated with thrombin to expose an N-terminal glycine, which was modified with a fluorescent peptide using SaSrtA. As the LPXTA sequence is not recognised by SaSrtA, this allowed for dual labelling of the UCHL3 protein and demonstrates orthogonal sortase labelling. This technique was also used for the N- and C-terminal labelling of an eGFP protein.

Hess *et al.* have also demonstrated orthogonal labelling with SpSrtA and SaSrtA by functionalising distinct capsid proteins in the same M13 bacteriophage particle. First, the N-terminus of pVIII was labelled with TAMRA-LPETGAA *via* SpSrtA, followed by N-terminal labelling of pIII with an antibody-LPETG *via* SaSrtA.^145^ The group then demonstrated triple capsid protein labelling (Scheme 5B),^141^ which was achieved by engineering a loop into pIII, containing a Factor Xa cleavage site and LPXTG motif. The first label, containing the LPETGAA motif, was attached to the N-terminus *via* SpSrtA. Cleavage with Factor Xa, revealed the LPXTG motif in the loop. SaSrtA could then be used to simultaneously label the pIII protein at the C-terminal site with a triglycine-containing substrate, along with pentaglycine installed at the N-terminus of pIX with a LPETGG-containing substrate.

The sortases SaSrtA and CdSrtA 3M have also been used for sequential site-specific dual labelling.^104^ These two enzymes are orthogonal as they recognise distinct nucleophiles, for SaSrtA an N-terminal glycine and for CdSrtA a lysine in a pilin motif. A fusion protein containing a SUMO protein with an N-terminal pentaglycine peptide, and a C-terminal pilin motif (G_5_-SUMO_PM_) was produced. The protein was first incubated with CdSrtA 3M and FITC-LPLTGpep to yield G_5_-SUMO_PM_-FITC through conjugation of the threonine of the peptide to the lysine in the pilin motif. After removal of excess FITC-LPLTG peptide using a desalting column, the target protein was then incubated with AlexaFluor546-LPATG and SaSrtA. The threonine of the peptide was conjugated to the N-terminal glycine of the protein, producing the double labelled product. The advantage of this approach is the distinct nucleophile and sorting signal substrate specificities of each sortase which limits cross reactivity. CdSrtA 3M is unable to hydrolyse the LPATG sequence or use it as a transpeptidation substrate; it is specific to LPLTG. Conversely, the isopeptide bond creating by CdSrtA 3M is not significantly hydrolysed by SaSrtA or CdSrtA after 24 hours.

Despite these advances using other natural sortases, there is currently only one example of orthogonal sortase-labelling with SaSrtA enzymes with altered specificity. This would be a superior approach as extensive investigations have been carried out on SaSrtA to understand the structure and mechanism, as well as engineering of the enzyme and substrates to generate efficient labelling strategies. Le Gall *et al.*^146^ used a CRISPR/Cas9 based strategy to engineer a hybridoma secreting mIgG1 antibodies (anti-CD20 WT) to a stable daughter cell line producing Fab′ fragments carrying two distinct sortase motifs; an eSrt2A-9 (LAETGG) motif on its heavy chain and an eSrt4S-9 (LPESGG) motif on its light chain (anti-CD20 DTFab′). The DTFab’ molecules could be isolated and modified at the sortase motif sites. Upon incubation with either sortase mutant, eSrt2A-9 or eSrt4S-9, in the presence of a GGC-C-K(FAM) peptide, exclusive fluorescent labelling was detected at the heavy chain (HC) or light chain (LC) labelling sites, respectively. Cross reactivity was not seen for either reaction, indicating that the close proximity of the sortase motif sites did not affect the specificity of either enzyme and allowed distinct payloads at each at the C-termini of the HC and LC. The researchers then demonstrated sequential dual site-specific modification by first incubating DTFab′ with eSrt4A-9 and GGG-C-K(FITC), achieving near quantitative labelling of the HC. Upon isolation of the labelled product, a 60% yield was achieved. Following this, the DTFab’FITC product was incubated with eSrt4S-9 and GGG-K(N_3_), achieving near quantitative labelling of the LC. The DTFab′FITC/N_3_ product could be isolated with a 50% yield. Further modification of the LC was achieved by reacting the azide group on the peptide with PEG_5k_-DBCO in a strain-promoted alkyne-azide cycloaddition (SPAAC). MALDI-TOF and SDS-PAGE was used to confirm the identity and purify of the final product. The target binding capacity of the obtained dual-labelled Fab’ fragment was not compromised. As a result, strategies such as this one could be valuable in the development of next-generation antibody–drug conjugates.

Although promising, the main downside to this technique is the large amounts of excess labelling reagent used (50 equivalents) If the reagent is precious, such as a cytotoxic payload, then this labelling strategy is not appropriate. However, a work around strategy of adding a functional group into the labelling reagent to enable a more efficient conjugation strategy (strain-promoted cycloaddition) to further modify the compound is possible, as utilised in this example. The dual labelling in this strategy must also be carried out sequentially. The ultimate goal would be to do these modifications in a one-pot reaction.

**Scheme 6:**
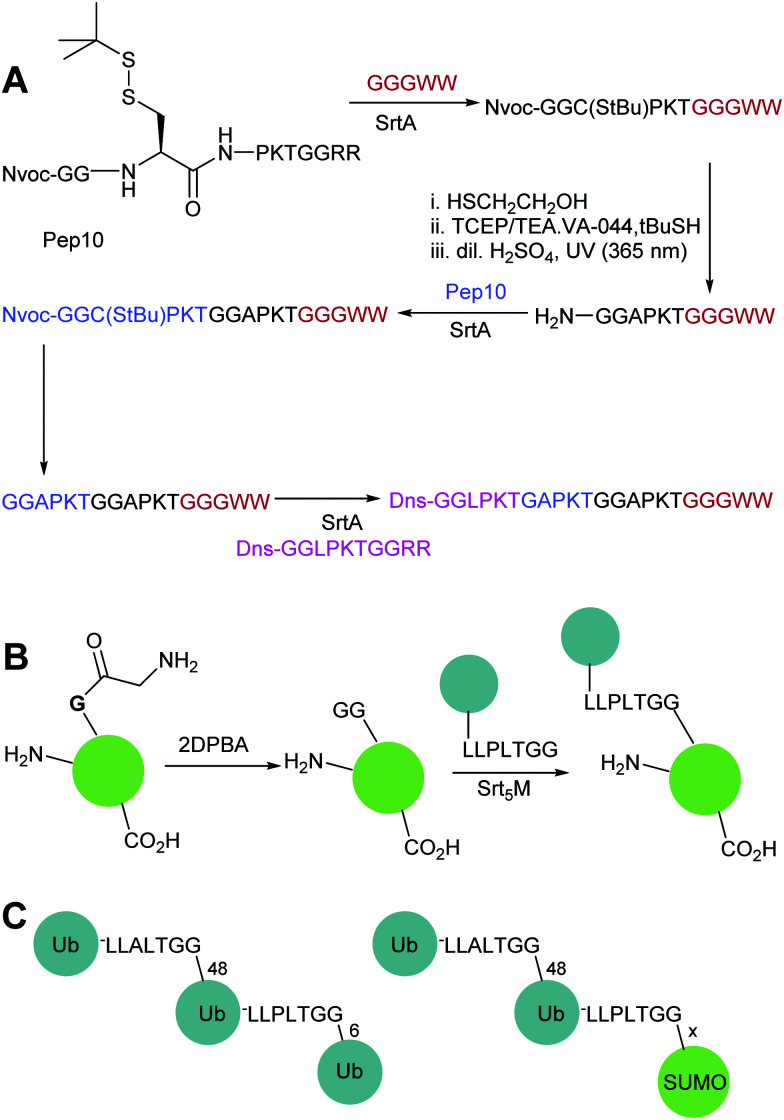
Recent examples of expansion of the substrates for peptide ligases to enable segment assembly and the generation of complex assemblies such as triubiquitins. (A) The use of tertbutylthiol cysteine disulfides as leucine isosteres enables the generation of sortase substrates which can then be deactivated by reduction and desulfurisation.^147^ (B) Incorporation of azidoacetyl glycyl lysine into proteins enables subsequent reduction using 2-diphenylphosphinobenzoic acid (2DPBA) and labelling using sortases.^148^ (C) Extension of this approach to applications with multiple orthogonal sortases enables the synthesis of specific triubiquitin and diubiquitylated SUMO constructs using both internal and N-terminal labelling.^149^

In an alternative approach, Bierlmeier *et al.* achieved orthogonal multi-fragment assembly with one enzyme, SaSrtA, *via* ligation site switching (Scheme 6A).^147^ The group identified that the leucine in the P4 position of the LPXTG motif could be replaced with l-Cys(StBu) and still be recognised by SaSrtA. Once this residue is reduced to cysteine (and further desulfurized to alanine), the motif is no longer recognised by the enzyme, switching it from an ON state to an OFF state. This approach was used in a proof of concept four fragment ligation reaction with a nucleophilic GGGWW peptide and Nvoc-GG-C(StBu)PKTGGRR. The GGGWW peptide was ligated to the C-terminus of the motif-containing peptide to produce ligation product Nvoc-GG-C(StBu)PKTGGGWW. Reduction and desulfurization of C(StBu) residue converted it to alanine and switched OFF the motif, preventing further C-terminal labelling of the peptide. The N-terminus could then be Nvoc-deprotected and further reacted with a sortase-motif containing peptide. The ligation site switching sortase-mediated ligation approach was also used to develop artificial nucleosome mimics to probe bivalent chromatic factors and antigen oligomers to probe antigen-presenting cell function.

In addition to direct anchoring of proteins to the cell surface, many sortases function to link proteins such as pilins together on the bacterial cell surface by covalently linking a sorting motif to a lysine residue within the protein. Despite this, attempts to exploit this reactivity have been limited and most of these enzymes show limited reactivity beyond their native substrate and yields for engineered motifs are typically low. Lang and co-workers^148^ have recently described an exciting approach which enables such conjugation by the use of genetic-code expansion to incorporate an ε-azidoacetyl-glycyl-lysine residue into the peptide backbone. Following Staudinger reduction to reveal a diglycyl motif, they were able to generate a range of diubiquitin analogues using both Srt5M and eSrtA(2A-9) *in vitro* in addition to site-specifically SUMOlyated and ubiquitylated proteins (Scheme 6B). Most excitingly, they were able to carry out both the reduction using 2-(diphenylphosphino)benzoic acid and the sortase-modification step in both *E. coli* and mammalian cells using a Ca-independent variant of eSrtA(2A-9). They have subsequently expanded this work and demonstrated that the eSrtA(2A-9) and Srt5M are orthogonal enabling them to generate a range of complex tri-ubiquitin and mixed ubiquitin/SUMO scaffolds (Scheme 6C).^149^

### 5.2 Butelase-1

**Scheme 7:**
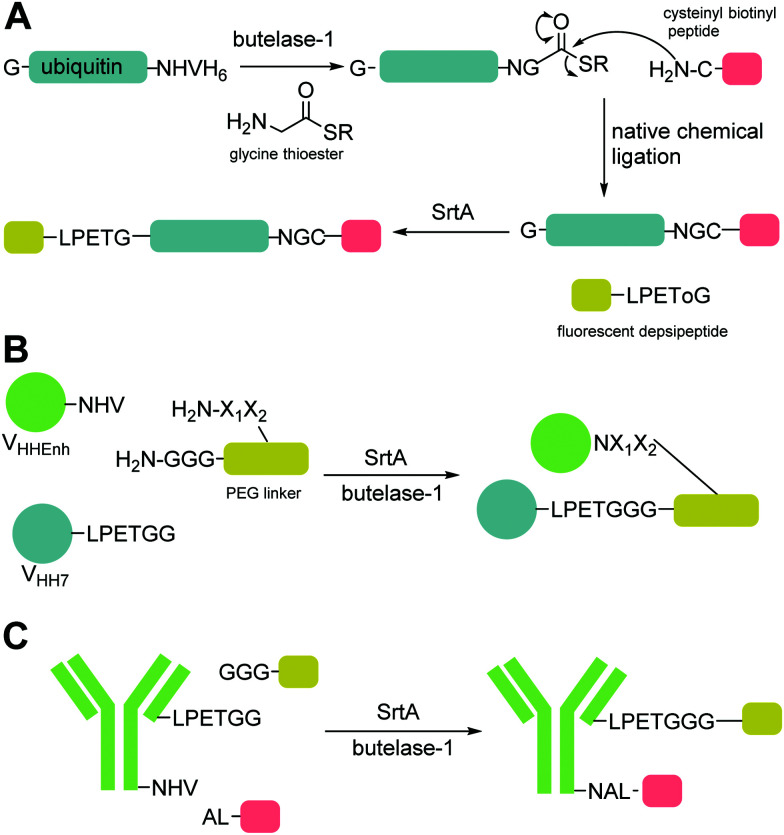
Examples of the combined application of SrtA and butelase-1 to enable double labelling of proteins and formation of protein fusions. Orthogonal labelling combining SaSrtA and butelase-1. (A) dual labelling of ubiquitin *via* a three-step tandem ligation with native chemical ligation.^150^ (B) One-pot conjugation of two nanobodies *via* their C-termini to produce C-to-C protein conjugates.^151^ This was done with a PEG linker and oligonucleotide linker. (C) One-pot conjugation at the C-terminus of the light chain and heavy chain of an antibody.^151^

Sortase A has also been in used in combination with butelase-1 to perform multiple ligations onto a protein/peptide. This has been demonstrated by Cao *et al.*^150^ who performed dual-terminal labelling of a protein using a three-step tandem ligation (Scheme 7A). For this approach, an engineered ubiquitin with an N-terminal glycine and C-terminal NHV-His_6_ motif was reacted with a glycine thioester in a C-terminal butelase-mediated ligation to produce a protein thioester. A cysteinyl biotinyl peptide was then ligated to the thioester to demonstrate that butelase-1 ligation can be used to prepare a protein thioester, useful for native chemical ligation (NCL). Finally, N-terminal sortase-mediated ligation was used to conjugate a fluorescent LPEToG depsipeptide to the N-terminus of the ubiquitin protein.

Due to their recognition of different motifs, sortase A and butelase-1 can be used for multiple labelling of a protein in a one-pot reaction, reducing reaction times and increasing product yield.^85^ This was demonstrated by Harmand *et al.*^151^ who conjugated two V_HH_s (nanobodies) *via* their C-termini to produce C-to-C protein conjugates (Scheme 7B). One nanobody, *V*_HH7_, contained the LPETGG motif and the other, *V*_HH–Enh_, contained the NHV motif. These proteins were conjugated through a two-headed PEG-based linker *via* sortase-mediated and butelase-mediated ligation, respectively. Another conjugate was produced in a similar fashion with a double-stranded oligonucleotide as a linker, leading to a protein–DNA–protein product. In the same paper, one-pot orthogonal dual labelling was used to produce an antibody-probe conjugate (Scheme 7C). Orthogonal butelase-1 and sortase A were utilised to modify a full-size antibody IgG1 at the C-terminus of the light chain and heavy chain, respectively, *via* their recognition motifs engineered into the molecules.

### 5.3 VyPAL2

**Scheme 8:**
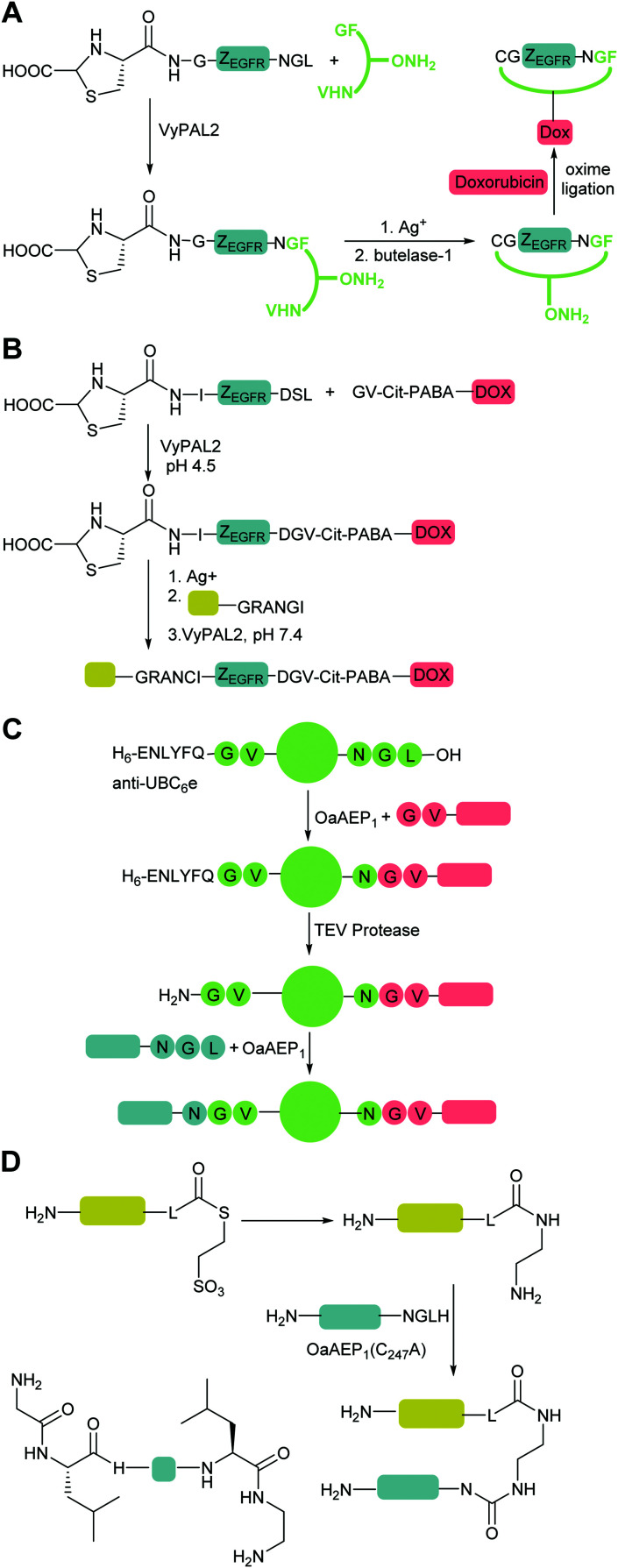
Examples of the use of P (A) orthogonal labelling combining butelase-1 and VyPAL2 to prepare a cycloprotein-drug conjugate.^152^ (B) pH controlled orthogonal ligation with VyPAL2 to produce a fluorescein-drug-labelled affibody.^153^ (C) Substrate controlled orthogonal labelling of an anti-UBC6e nanobody *via* OaAEP1^91^ (D) Use of C-terminal 2-aminoethylamides to enable C–C tail-to-tail protein dimerisation using OaAEP1. General structure of peptide substrates for homodimerisation and strategy to enable heterodimerisation *via* use of C-terminal protein thioesters.^154^

Butelase-1 has also been used in combination with VyPAL2 for orthogonal ligation. Wang *et al.*^152^ used kinetic and structural analysis to confirm that butelase-1 is tolerant of a range of residues at the P1′ but preferred bulky aliphatic chains such as Val at P2′ due to the presence of a Val residue in the S2′ pocket. In contrast, VyPAL2 the S1′ pocket of VyPAL2 is sterically restricted by an alanine residue and a lysine residue in the S2′ pocket favours binding of larger residues such as Ile and Phe. Based on this, they developed two distinct NHV and NGF/NGI motifs for use with butelase-1 and VyPAL2, respectively. They demonstrated the application of this approach to tandem labelling of an affibody (*Z*_EGFR_) with an N-terminal GF dipeptide and C-terminal NHV tripeptide. The N-terminus of the protein was labelled with a fluorescein-NGI peptide and VyPAL2 followed by C-terminal labelling with a GI-KLA motif peptide and butelase-1. This second step led to removal of a small amount (10%) of the N-terminal label due to some cross-reactivity of butelase-1 with the NGF motif. Carrying out the reactions in the opposite order led to a cleaner product without significant reaction between VyPAL2 and the NHV motif. The same approach was used to prepare a cycloprotein-drug conjugate (Scheme 8A). A trifunctional peptide containing an N-terminal GF-dipeptide nucleophile substrate for VyPAL2, a C-terminal NHV tripeptide motif as the acyl donor substrate for butelase-1 and an internal aminooxy functionality for oxime conjugation was used with a recombinant *Z*_EGFR_ with an N-terminal CG motif and C-terminal NGL motif. The N-terminal cysteine residue was capped as a thiazolidine during protein production, blocking it from being used as a nucleophilic substrate by either butelase-1 or VyPAL. Thus, only the C-terminally labelled *Z*_EGFR_ product was generated in the first ligation step *via* VyPAL2 without the possibility of cyclisation or self-ligation. The cysteine was then unmasked *via* treatment with silver nitrate and β-mercaptoethanol. Butelase-mediated ligation of the C-terminal NHV motif and N-terminal CG motif produced the cyclised *Z*_EGFR_ product. The aminoxy-functional group was then conjugated to doxorubicin ketone group using aniline catalysis.

The same group, led by Zhang,^153^ have subsequently used VyPAL2 alone for sequential orthogonal ligation by controlling the pH of the reactions. All previous applications of PALs, described above, involve the use of 
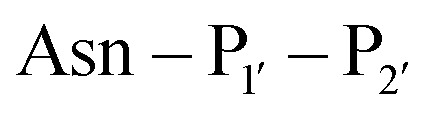
 substrates only, likely due to their enhanced ligation efficiency at neutral pH compared to their aspartic acid-containing counterparts.^72–75^ VyPAL2 has already been shown to preferentially bind Leu in the 
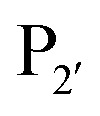
 position.^81^ Zhang *et al.* showed that VyPAL2 worked effectively on peptides with the sequence DSL with a pH optimum of pH 4.5 (presumably driven by the balance of the need for protonation of the aspartic acid in the S1 pocket). In contrast, VyPAL2 catalyses ligation reactions at neutral pH most efficiently with NGL-containing peptides.^81^ They therefore used pH to switch the selectivity of VyPAL2 and two separate substrates by altering the pH of the reaction. An sfGFP protein was produced with an N-terminal GV and C-terminal NSL was labelled on the N-terminus using a C-terminal DSK DSL-containing targeting peptide at pH 4.5. After purification, C-terminal ligation with a N-terminal GV-containing DOX peptide was carried out at neutral pH; the DGV motif formed in the first reaction was unaffected. A one-pot tandem ligation was also achieved with an adjustment of pH after the addition of the second substrate though in this case a side reaction between the two labelling substrates was observed. To carry out labelling in the opposite order, an affibody (*Z*_EGFR_) was prepared with a C-terminal DSL sequence, and a thiazolidine-capped CI motif at the N-terminus to prevent cyclisation (Scheme 8B). The first reaction with GV-Cit-PABA-DOX was carried out at pH 4.5 followed by deprotection of the N-terminus with Ag^+^ to allow ligation of asparagine-containing Fluorescein-GRA**NGI** at an adjusted pH of 7.4. Using OaAEP1 for this ligation led to formation of a significant quantity of a cyclisation product as the DG bond formed in the first ligation is not completely orthogonal to OaAEP1 at pH 7.4. However, OaAEP1 has higher catalytic activity towards aspartic acid-containing substrates than VyPAL2 and butelase-1. Thus, use of OaAEP1 for the first ligation step and VyPAL2/butelase-1, which show optimum activity towards asparagine-containing substrates at neutral pH, for the second step produced a dual-fluorescein-labelled affibody with higher efficiency and specificity.

### 5.4 OaAEP1

OaAEP1 has also been used on its own for orthogonal ligation. As discussed above, upon the discovery by Rehm *et al.*^91^ that GV-based nucleophiles are readily ligated by OaAEP1 C247A, yet the resultant NGV ligation product is a poor substrate, the group then explored the use of the technique to perform site-specific sequential ligation reactions on a single protein. This negated the requirement for multiple orthogonal substrate specific enzymes. The group demonstrated this by using OaAEP1 C247A to N- and C-terminally dual label an anti-UBC6e nanobody (Scheme 8C). The nanobody was prepared with a TEV-recognition sequence (ENLYFQ) at the N-terminus followed by the GVGS sequence and a C-terminal NGL. In the first reaction, the C-terminus of the protein was labelled with a GV-nucleophile, generating the NGV sequence. To reveal the GV-nucleophile at the N-terminus, TEV cleavage was performed. The N-terminus was then site-specifically labelled with an NGL probe, leaving the C-terminal NGV sequence intact. At each step, >90% conversion was achieved, with only minimal purification required to remove enzyme and probe between steps.

More recently the relatively short recognition motif of OaAEP1 has been exploited by the same group to enable the synthesis of C–C tail-to-tail dimeric proteins.^154^ To generate homodimeric proteins they generated synthetic peptide substrates with an N-terminal GLH motif and a C-terminal leucyl-ethylene diamine motif which mimics the N-terminal GL motif (Scheme 8D). This could then be used with OaAEP1 to generate homodimeric proteins. A variety of candidate amino-acids other than l-leucine were explored and in general the l-enantiomers were preferred to d- and leucine to valine. For heterodimeric proteins, an intein-based strategy was used to generate one-target protein with a C-terminal leucyl thioester. Aminolysis with ethylene diamine generated a C-terminal amine which is then sufficiently close to the normal N-terminal GlyLeu motif that OaAEP recognises it and is able to catalyse transpeptidation to a protein bearing a C-terminal NGLH motif.

### 5.5 Subtiligase

Following on from the labelling reactions to assess the ability of engineered subtiligase mutants to modify a recombinant αGFP antibody at the N-terminus of the heavy and light chain, as described above, Weeks and Wells^107^ next explored whether orthogonal labelling of αGFP could be achieved. As the light chain was not quantitatively labelled in the previous experiment, the N-terminus was expanded by Ala–Phe–Ala, a sequence favourable for WT subtiligase. Specific and quantitative labelling of the light chain was achieved within 1 hour with WT subtiligase. When using subtiligase-Y217K, labelling of both the heavy chain and light chain occurred. These results demonstrate that careful selection of subtiligase mutants matched to their optimal substrates by PILs can lead to orthogonal labelling. However, a subtiligase mutant that modifies a single, specific sequence has yet to be engineered.

## 6 Conclusions

Controlled protein modification is a widely applied technique and the peptide ligases discussed in this review have been a critical part of these developments. In the future, these approaches have the potential to enable the generation of ever more complex multiply-modified proteins and to generate multimeric proteins from multiple protein building blocks. However the three principal challenges in developing new methods highlighted in the introduction still apply: the reactions need to be site-specific and generate well-defined conjugates; the enzymes need to be versatile and readily applied to other contexts; and finally that the methods need to be time and reagent efficient. While significant progress has been made to date, major challenges remain before the methods can be generally applied to enable routine synthesis of complex architectures such as those most recently exemplified by *e.g.* Lang and coworkers.^149^

The sortases and the peptidyl asparaginyl ligases such as Butelase and OaAEP1 show the greatest promise for future applications in protein engineering. While ligases generated from proteases such as subtiligase have potential, their requirement for ester and thioester substrates and low specificity makes their application in protein engineering more challenging. In the case of sortase, rounds of protein engineering mean there are now a wide range of enzymes with increased rate of reaction as well as mutual orthogonality. These enzymes are often readily available with high bacterial overexpression yields. In contrast, PALs are not yet as readily available but are faster and have shorter recognition motifs which may be better tolerated in the final protein products.

In this regard, the first challenge addressed in the introduction, specificity, has been addressed with a number of sortases and peptidyl asparaginyl ligases now available with distinct recognition motifs. Doubtless this range will be increased in the coming years, *via* directed evolution and the discovery and characterisation of other naturally occurring peptidyl asparaginyl ligases and sortases. This field is of particular interest, since numerous other housekeeping sortases are extant in Gram-positive bacteria. If we can understand how they recognise their protein substrates then it will be possible to develop a new class of reagents which label, for example, internal lysine residues in defined sequence motifs.

The second challenge, versatility, has been demonstrated most clearly for the sortases. Ca-independent variants have enabled in cell labelling and the application of diverse variants has now started to enable the synthesis of complex protein scaffolds as well as their successful applications in cells. Despite these successes, challenges remain: currently the peptidyl asparaginyl ligases have only really been applied to *in vitro* systems and approaches for their recombinant expression are only now being optimised. The exploitation of these and other related PALs in combination with sortases promises to be a rich area of development for complex protein and peptide assembly.

The final challenge, efficiency, is critical for the wider application of these approaches. Since transpeptidation is an equilibrium process, driving reactions to completion almost inevitably requires an excess of reagents. This is particularly noticeable in most described examples of protein fusion where an excess of one protein component is required. Even when optimised peptide substrates for labelling are used, an excess is usually required. Numerous approaches to perturb these equilibria such as the use of depsipeptide substrates and substrates which form complexes with metals or other small molecules, or the use of mechanical separation to remove low molecular weight by-products have been reported. All these approaches carry challenges however, from the requirement to add divalent metals to the protein, to the need to generate complex synthetic substrates – ultimately in all these cases a moderate excess of one reagent is still required and no approach in which essentially quantitative ligation using 1 : 1 reagents has been reported. The major challenge to enabling such a ‘perfect’ reaction, in which two substrate molecules are ligated to generate the desired product, remains the hydrolysis side-reaction. Most peptide ligases such as sortase also catalyse hydrolysis of their substrate although the aminolysis reaction is approximately 10^5^–10^6^-fold favoured over hydrolysis at optimal pH. Despite this selectivity, excess or high concentrations of labelling reagents are needed to compensate for their hydrolysis, or more critically, the hydrolysis of protein substrates which makes them incompetent for subsequent labelling reactions. Whether it is possible to evolve ligases to avoid this challenge remains to be seen but this and the development of other strategies to maximise product formation will be critical if the use transpeptidases are to be extended from single labelling reactions to the efficient synthesis of large multicomponent assemblies in a routine fashion, and even on industrial scale.

## Conflicts of interest

There are no conflicts to declare.

## Supplementary Material
